# Effects of Technology Assisted Stepped Collaborative Care Intervention to Improve Symptoms in Patients Undergoing Hemodialysis: The TĀCcare Randomized Clinical Trial

**DOI:** 10.1001/jamainternmed.2023.2215

**Published:** 2023-06-20

**Authors:** Manisha Jhamb, Jennifer L. Steel, Jonathan G. Yabes, Maria-Eleni Roumelioti, Sarah Erickson, Susan M. Devaraj, Kevin E. Vowles, Yoram Vodovotz, Scott Beach, Steven D. Weisbord, Bruce L. Rollman, Mark Unruh

**Affiliations:** 1 Renal-Electrolyte Division, Department of Medicine, University of Pittsburgh School of Medicine, Pittsburgh, Pennsylvania; 2 Department of Surgery, Psychiatry and Psychology, University of Pittsburgh, Pittsburgh, Pennsylvania; 3 Center for Research on Heath Care Data Center, Division of General Internal Medicine, Department of Medicine and Biostatistics, University of Pittsburgh, Pittsburgh, Pennsylvania; 4 Division of Nephrology, Department of Internal Medicine, University of New Mexico School of Medicine Albuquerque; 5 Department of Psychology, University of New Mexico, Albuquerque; 6 School of Psychology, Queen’s University, Belfast, Northern Ireland, United Kingdom; 7 Department of Surgery, University of Pittsburgh, Pittsburgh, Pennsylvania; 8 Department of Psychology, University Center for Social and Urban Research, University of Pittsburgh, Pittsburgh, Pennsylvania; 9 Center for Behavioral Health, Media, and Technology, Division of General Internal Medicine, University of Pittsburgh, Pittsburgh, Pennsylvania; 10 Renal Section and Center for Health Equity Research and Promotion, Veterans Affairs Pittsburgh Healthcare System, Pittsburgh, Pennsylvania

## Abstract

**Importance:**

Patients with end-stage kidney disease (ESKD) undergoing long-term hemodialysis often experience a high burden of debilitating symptoms for which effective treatment options are limited.

**Objective:**

To compare the effectiveness of a stepped collaborative care intervention vs attention control for reducing fatigue, pain, and depression among patients with ESKD undergoing long-term hemodialysis.

**Design, Setting, and Participants:**

Technology Assisted Stepped Collaborative Care (TĀCcare) was a parallel-group, single-blinded, randomized clinical trial of adult (≥18 years) patients undergoing long-term hemodialysis and experiencing clinically significant levels of fatigue, pain, and/or depression for which they were considering treatment. The trial took place in 2 US states (New Mexico and Pennsylvania) from March 1, 2018, to June 31, 2022. Data analyses were performed from July 1, 2022, to April 10, 2023.

**Interventions:**

The intervention group received 12 weekly sessions of cognitive behavioral therapy delivered via telehealth in the hemodialysis unit or patient home, and/or pharmacotherapy using a stepped approach in collaboration with dialysis and primary care teams. The attention control group received 6 telehealth sessions of health education.

**Main Outcomes and Measures:**

The coprimary outcomes were changes in fatigue (measured using the Functional Assessment of Chronic Illness Therapy Fatigue), average pain severity (Brief Pain Inventory), and/or depression (Beck Depression Inventory-II) scores at 3 months. Patients were followed up for 12 months to assess maintenance of intervention effects.

**Results:**

There were 160 participants (mean [SD] age, 58 [14] years; 72 [45%] women and 88 [55%] men; 21 [13%] American Indian, 45 [28%] Black, 28 [18%] Hispanic, and 83 [52%] White individuals) randomized, 83 to the intervention and 77 to the control group. In the intention-to-treat analyses, when compared with controls, patients in the intervention group experienced statistically and clinically significant reductions in fatigue (mean difference [md], 2.81; 95% CI, 0.86 to 4.75; *P* = .01) and pain severity (md, −0.96; 95% CI, −1.70 to −0.23; *P* = .02) at 3 months. These effects were sustained at 6 months (md, 3.73; 95% CI, 0.87 to 6.60; *P* = .03; and BPI, −1.49; 95% CI, −2.58 to −0.40; *P* = .02). Improvement in depression at 3 months was statistically significant but small (md −1.73; 95% CI, −3.18 to −0.28; *P* = .02). Adverse events were similar in both groups.

**Conclusions and Relevance:**

This randomized clinical trial found that a technology assisted stepped collaborative care intervention delivered during hemodialysis led to modest but clinically meaningful improvements in fatigue and pain at 3 months vs the control group, with effects sustained until 6 months.

**Trial Registration:**

ClinicalTrials.gov Identifier: NCT03440853

## Key Points

**Question:**

Does a stepped collaborative care intervention including psychotherapy or pharmacotherapy delivered via telehealth improve symptoms of fatigue, pain, or depression among patients with end-stage kidney disease (ESKD) who are undergoing long-term hemodialysis?

**Findings:**

This randomized clinical trial of 160 patients with ESKD undergoing hemodialysis with at least moderate levels of fatigue, pain, or depression found that treatment with a stepped collaborative care intervention delivered during hemodialysis or at home vs health education control resulted in clinically significant improvements in fatigue and pain.

**Meaning:**

These findings show that this stepped collaborative care intervention is a significantly effective treatment for fatigue and pain in patients undergoing long-term hemodialysis for ESKD.

## Teaser

This randomized clinical trial evaluates the effects of a stepped collaborative care intervention delivered during hemodialysis to improve pain, fatigue, and depression in patients with end-stage kidney disease.

## Introduction

Patients with end-stage kidney disease (ESKD) undergoing treatment with long-term hemodialysis experience a high symptom burden that contributes to poor health-related quality of life (HRQOL).^1,2,3,4^ The most common and debilitating symptoms include fatigue, pain, and depression, which have been reported by more than 70%, 50%, and 20% of patients undergoing dialysis, respectively; these are often underrecognized, underreported, and inadequately treated.^5,6,7^ Identifying effective ways of managing symptoms is a top priority for patients and dialysis care partners.^8,9^ Recently, the Kidney Disease Improving Global Outcomes organization advocated for integration of symptom assessment and management into routine ESKD care and more research into treatment strategies.^10^

Currently available treatments for these symptoms are limited for patients receiving hemodialysis. The most promising results have been for treatment of depression using pharmacotherapy or psychotherapy.^11,12,13^ Limited data suggest there are benefits of analgesic medications for pain; and of sleep hygiene, exercise, and anemia correction for fatigue.^14,15,16^ However, methodologic limitations of most studies, mixed results on efficacy, adverse events, pill burden associated with pharmacotherapy, and lack of inclusion of robust fatigue- or pain-specific cognitive behavioral therapy (CBT) underscore the need for alternative strategies.^16,17^ Moreover, prior studies have largely focused on treatment of a single symptom, whereas treatment of *symptom clusters* may be more effective given that many of the physical and mental symptoms frequently coexist, are highly correlated, can exacerbate each another, and may share similar biologic and psychologic pathogenesis.^18,19^

To address these gaps in symptom management, we designed the Technology Assisted Stepped Collaborative Care (TĀCcare) intervention to target a common symptom cluster—fatigue, pain, and depression—and used a flexible approach to offer clinical treatment options that were tailored to individual preferences and treatment responses. Either CBT and/or pharmacotherapy can be initiated sequentially, and subsequently, both psychotherapy and medication dosages may be escalated in a stepped fashion using evidence-based protocols. Additionally, a collaborative model was incorporated to promote care coordination among dialysis and primary care teams. The goal of our study was to compare the effectiveness of the TĀCcare intervention with an attention control in improving fatigue, pain, and depression among patients with ESKD who are undergoing long-term hemodialysis.

## Methods

### Study Overview

This was a 2-site, parallel group, randomized clinical trial (RCT) that compared results of the TĀCcare intervention with those of an attention control (health education) intervention in improving key patient-centered outcomes among 160 patients receiving long-term hemodialysis in New Mexico and Western Pennsylvania from March 2018 to June 2022 (additional details are available in the Trial Protcol in Supplement 1). The study was approved by the institutional review boards of both sites and was overseen by an external data safety and monitoring board and adverse events committee. All participants provided written informed consent. Results were reported according to the Consolidated Standards of Reporting Trials (CONSORT) reporting guideline. Additional information on the study protocol was published previously.^20^

### Setting and Participants

Adult patients (≥18 years) receiving in-center 3-times-weekly maintenance hemodialysis from 6 dialysis units in Western Pennsylvania and 8 dialysis units in New Mexico were recruited for study participation. Participants were enrolled from March 1, 2018, to Dec 31, 2021, and were followed uo through June 31, 2022. All patients were asked to complete a screening for fatigue (Likert scale, 0-10 with higher score indicating worse fatigue), pain (Likert scale, 0-10 with higher score indicating worse pain), and depression (Patient Health Questionnaire-9, higher score indicating worse depression) experienced during the past 2 weeks.^21^ To minimize patient burden, we used these single-item questions for fatigue and pain screening adapted from validated questionnaires, with cutoffs for a clinically significant level of symptoms based on literature review (≥5 for fatigue; ≥4 for pain; ≥10 on the Patient Health Questionnaire-9).^22,23,24,25^ Participants with clinically significant levels of 1 or more of the 3 symptoms were screened for readiness for treatment using the 5-item Stages of Behavior Change questionnaire.^26^ We approached patients who were at least in the contemplation stage of behavioral change for trial consent (ie, those who responded “would be motivated to seek treatment or talk to doctor about it”). We excluded those who had evidence of active thought disorder, delusions or active suicidal intent, active substance abuse, cognitive impairment, and anticipated life expectancy of less than 1 year, were scheduled for a living donor kidney transplant, or who planned to relocate to another dialysis unit within 6 months. We did not exclude patients currently receiving treatment for pain or depression because studies suggest that these symptoms are often undertreated in patients receiving hemodialysis.^27^

**Figure 1.**
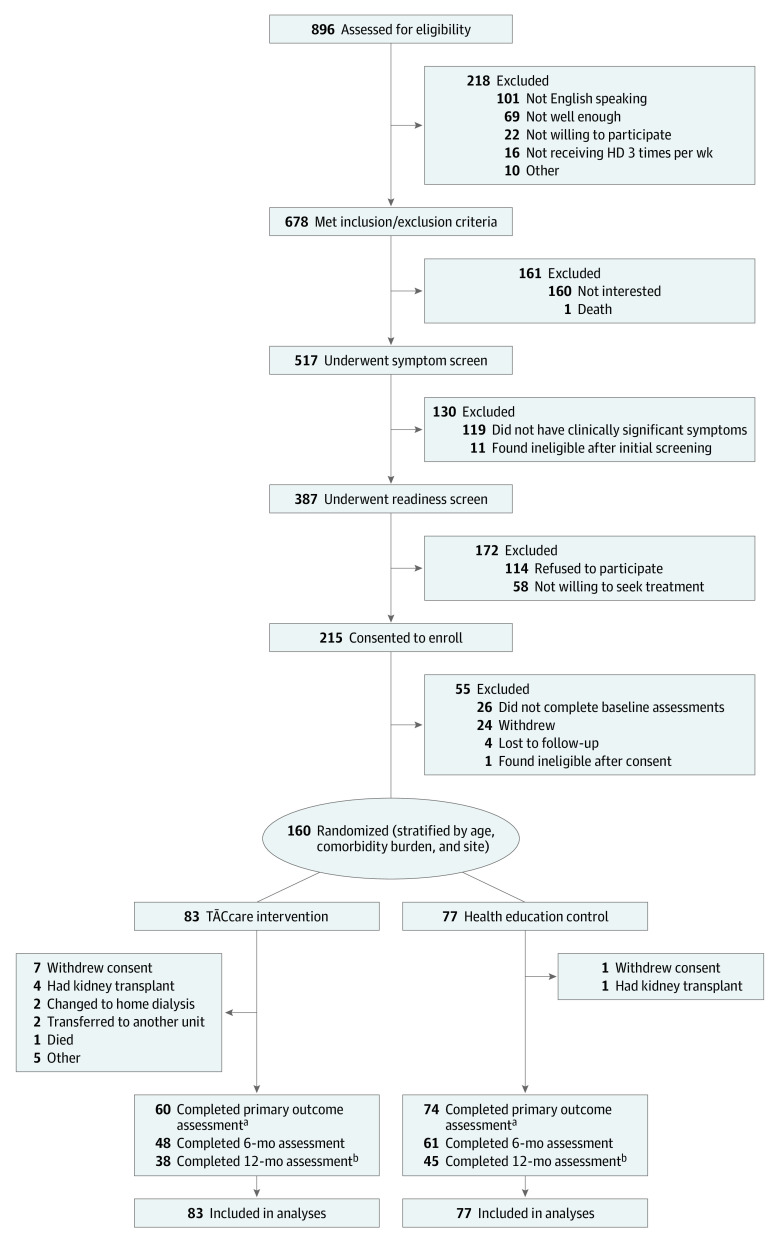
CONSORT Flow Diagram of Participant Enrollment in the TĀCcare Randomized Clinical Trial ^a^Two patients in the intervention group and 1 patient in the control group did not complete the 12-week intervention but continued participation in the remaining study. ^b^Six patients in the intervention and 7 patients in the control group did not reach the 12-month time point because data collection was terminated owing to the COVID-19 pandemic restrictions. HD refers to hemodialysis.

Of the 896 patients screened, 678 (76%) met eligibility criteria, and 517 (76%) completed screening for symptom severity and readiness to seek treatment. Of the 387 patients (75%) who had clinical levels of at least 1 symptom (fatigue, pain, or depression) and were at least in the contemplation phase of readiness to seek treatment, 215 (65%) consented to participate, and 160 (74%) of them were randomized (Figure 1).

### Randomization and Blinding

Using a priori computer-generated permuted block design with random block sizes stratified by the 2 clinical sites, age (<60 or ≥60 years), and Charlson Comorbidity Index (CCI) score (<4 or ≥4), we randomized the participants in a 1:1 ratio to either the intervention or the control group. A central data manager conducted treatment assignments from a pregenerated random allocation sequence and informed the local research staff, who subsequently conducted a study visit with the participants to inform them of their treatment group. Although patients were not blinded given the nature of the study intervention, research staff assessing outcomes over telephone were blinded to treatment group.

#### Intervention Group

Participants randomized to the TĀCcare group received treatment targeted at 1 or more symptoms (fatigue, pain, and/or depression) based on patients’ reported levels of each symptom and preference. Using an individualized and shared decision-making approach, pharmacotherapy, and/or CBT were offered to patients for a 12-week period. A stepped approach to treatment intensification allowed for monitoring patient adherence, treatment response, preferences, and outcomes, and modifying the treatment to achieve the best possible outcome for each patient. The CBT strategies were contextualized to address the unique challenges and needs of each patient receiving hemodialysis.^20^ The therapists had a master’s degree in counseling or psychology, were trained and certified in CBT by a doctoral level psychologist, and received training on dialysis-related factors that affect symptom burden and management. They worked under the supervision of a local clinical psychologist with weekly case meetings and regular fidelity checks. To simplify the delivery of CBT and reduce patient and clinician burden, CBT was delivered using telemedicine in dialysis units or at home. The study provided preprogrammed iPads, portable secure Wi-Fi hotspots, wireless headphones, and microphones.

If patients preferred pharmacotherapy or did not respond to CBT, medications for pain and/or depression were initiated, or dosages were escalated in a stepped fashion using clinically used evidence-based protocols. There was no pharmacotherapy option for fatigue given the lack of any approved evidence-based fatigue medications for this population. The research team’s medication recommendations were conveyed to the patient’s primary care physician and/or nephrologist by the therapists or the study nephrologist. The collaborative care model ensured a multidisciplinary approach to the patient’s physical and mental health by aligning symptom management with the overall plan of care. Therapists facilitated care coordination, medication management, and monitoring of adherence and adverse events by serving as liaisons among patients, dialysis teams, and other health care professionals.

#### Control Group 

We used an attention control group rather than a usual care group to limit the variability of factors and the biases that might influence the outcomes, enhance the interpretability of study results, and enable evaluation of the true effects of our intervention, rather than those from just participation or attention.^28^ Health education was chosen as an attention control because it is relevant to the needs and health of the participants but is unlikely to have a specific effect on the study outcomes. The health education control was designed to feasibly approximate the amount of time and attention received by the TĀCcare group so that control group participants would be engaged, and the study would avoid dropouts or selection biases. Trained research coordinators used educational materials from the National Kidney Foundation to provide ESKD-relevant education on relevant topics—kidney transplantation, heart health, immunizations, diet, travel—per patient preference via telemedicine delivered in the dialysis units or at home.

#### Study Duration and Follow-up

Participants in the TĀCcare group received 12 weekly sessions (45-60 minutes each) of CBT; the control group received 6 biweekly sessions (20-30 minutes each) of health education. If interruptions occurred, the intervention period was extended for up to 6 months. Participants were followed up for 12 months or until June 31, 2022 (end of the data collection period), death, or loss to follow-up (withdrawal from study, transfer to another dialysis clinic, change to peritoneal dialysis, or kidney transplantation).

### Outcomes

The coprimary outcomes were change in fatigue, as measured using the Functional Assessment of Chronic Illness Therapy-Fatigue (FACIT-F)^29^, with higher scores indicating less fatigue; pain severity, using the Brief Pain Inventory Short Form (BPI,^25^ average pain severity item score), with higher scores indicating worse pain; and/or depression, using the Beck Depression Inventory II (BDI-II)^30^, with higher score indicating worse depression) as compared with the control group from baseline to 3 months. All patient-reported outcome assessments were administered centrally by blinded interviewers using computer-assisted telephone interviewing. Secondary outcomes included change in pain interference, measured using the BPI Short form^25^; HRQOL, using the Medical Outcomes Study Short Form-12 and the US National Institutes of Health Patient Reported Outcomes Measurement Information System (PROMIS) Adult Global Health^31,32^; sleep quality, using the Pittsburgh Sleep Quality Index^33^; anxiety, using the Generalized Anxiety Disorder-7^34^ form; social support, using the Multi-dimensional Scale of Perceived Social Support^35^; physical activity, using the Physical Activity Scale for Elderly^36^; postintervention adherence to medications, diet, and hemodialysis treatments; and change in symptom scores at 6 and 12 months.

Prespecified serious adverse events (SAEs) included death, hospitalization or emergency department visit, bleeding requiring transfusion or hospitalization, medication overdose requiring an emergency department visit or hospitalization, and acute suicidal intent. The relatedness of adverse events and SAEs with study interventions, especially with medications prescribed for symptom management, was reviewed by study nephrologists and an external adverse events committee. Lastly, patient satisfaction surveys completed anonymously were collected at end of study participation.

### Statistical Analysis

The primary analyses were based on the intention-to-treat approach. The 3 symptom end points were considered coprimary outcomes by design. To address for multiple testing, we controlled for a false discovery rate (FDR) of 5% using the Benjamini-Hochberg correction.^37^ For each end point, we used all available longitudinal data to fit linear mixed models with treatment group (TĀCcare or health education), time (baseline, 3, 6, and 12 months), and group by time interaction with patient-specific random intercepts. More details are available in the eMethods in Supplement 2.

After assessing the model diagnostics, we included quadratic time, its interaction with the treatment group, and random time slopes. To improve precision, these models were adjusted for prespecified covariates (age, sex, site, and CCI) that were chosen based on prior literature demonstrating an association with symptom burden or response to treatment.^38,39,40^ To estimate the treatment effect, a linear contrast was used to compare the changes in outcomes between groups at the 3-month primary time point. As an alternative model that accounts for potential correlation among symptoms and allows simultaneously testing intervention effects across the 3 primary outcomes, we also fitted a joint longitudinal model. This included the same fixed- and random-effects terms from the individual models with outcome specific regression coefficients and random effects, unstructured correlation between the random effects, and heteroscedastic residual error terms across the outcomes. To explore heterogeneity of intervention effects, prespecified subgroup analysis was used based on age, sex, race, CCI, and time on dialysis (years). Each variable, its 2- and 3-way interaction with the treatment group, and interaction with time were added to the model, and linear contrasts were used to test for heterogeneity in treatment effects. Additional subgroup analyses restricted to participants with clinically significant levels of fatigue (FACIT-F ≤44 [the US population mean])^41,42,43^, moderate to severe pain (BPI ≥5)^25,43^ and depression (BDI ≥16)^30^ at baseline were conducted.

Statistical analyses were conducted using R, version 4.2.1 (The R Foundation for Statistical Computing) with Ime4^44^ and MarginalEffects^45^ packages to fit the mixed models and perform the contrasts, and the nlme and emmeans packages^46^ for the joint longitudinal model analyses. We initially determined that 150 participants would provide 88% power to detect an effect size of 0.6, assuming 5% FDR for 3 end points with a true difference in 1 of 3 end points and 10% attrition. In May 2020, on the recommendation of the external data safety and monitoring board, the sample size was increased to 160 participants to address the higher observed attrition rate (14%). Statistical tests were 2-tailed and *P* values < .05 were considered statistically significant. Data analyses were performed from July 1, 2022, to April 10, 2023.

## Results

### Baseline Characteristics

The 160 participants (mean [SD] age, 58 [14] years; 72 [45%] women and 88 [55%] men; 21 [13%] American Indian or Alaska Native, 45 [28%] Black; 28 [18%] Hispanic, and 83 [52%] White individuals) who were randomized were of similar age, sex, and race or ethnicity compared with those not randomized after consent. Primary outcome assessment at 3 months was completed for 134 (84%) of the randomized patients.

Among the 160 randomized patients , 152 (94%) had fatigue, 74 (46%) had moderate-severe pain, and 69 (43%) had depression at baseline (eFigure and eTable 1 in Supplement 2). Baseline fatigue, pain, depression levels, and use of opioids had acceptable balance between groups; however, use of antidepressants was higher in the intervention group (Table 1). Baseline levels of other patient-reported outcomes and adherence to medications, diet, fluid restriction, and hemodialysis treatments did not differ between study groups (eTable 2 in Supplement 2).

**Table 1.** Baseline Characteristics of Patients Randomized in the TĀCcare Trial

Characteristic	TĀCcare intervention, No. (%)	Attention control, No. (%)	Absolute standardized bias (%)
Participants, No.	83	77	NA
Age, mean (SD), y	57.9 (14.0)	57.8 (13.7)	0.7
Female	34 (41.0)	38 (49.4)	16.9
Male	49 (59.0)	39 (50.7)	16.9
Race and ethnicity^a^			
American Indian/Alaska Native	7 (8.4)	14 (18.2)	29.2
Black	26 (31.3)	19 (24.7)	14.7
Hispanic (yes)	19 (22.9)	9 (11.7)	29.9
White	42 (50.6)	41 (53.2)	5.2
Other (>1 race/unknown)	8 (9.6)	3 (3.9)	22.9
Missing data	4 (4.8)	3 (3.9)	NA
Education ≥high school	72 (86.7)	69 (89.6)	9.0
Married	26 (31.3)	17 (22.1)	20.9
Employed	3 (3.6)	6 (7.8)	18.2
Tobacco use (ever)	45 (54.2)	41 (53.2)	2.0
Alcohol use (yes)	14 (16.9)	11 (14.3)	7.2
Household income <$40 000/y	60 (72.3)	62 (80.5)	19.4
Missing data	10 (12.0)	7 (9.1)	NA
Diabetes	50 (60.2)	51 (66.2)	12.5
Cardiovascular disease	39 (47.0)	28 (36.4)	21.6
CCI, mean (SD)	4.7 (1.7)	4.8 (1.8)	5.7
Cause of ESKD			
Diabetic nephropathy	37 (44.6)	41 (53.2)	17.3
Hypertensive nephrosclerosis	16 (19.3)	10 (13.0)	17.2
Other	27 (32.5)	23 (29.9)	5.6
Missing	3 (3.6)	3 (3.9]	NA
Time on dialysis, mean (SD), y	4.5 (4.6)	3.7 (3.6)	19.4
EDW, mean (SD), kg	85.9 (27.8)	89.5 (28.1)	12.9
Missing data	1 (1.2)	0	NA
IDWG, mean (SD), %	2.9 (1.2)	2.5 (1.1)	34.7
Psychotherapy during 6 mo before	9 (10.8)	10 (13.0)	6.8
Hemoglobin, mean (SD), g/dL	11.3 (1.3)	11.1 (1.4)	14.8
Phosphorus, mean (SD), mg/dL	5.6 (1.5)	5.6 (1.6)	0
Albumin, mean (SD), g/dL	4.0 (0.3)	3.9 (0.4)	28.3
Single pool, mean (SD), Kt/V	1.6 (0.3)	1.6 (0.5)	0
Creatinine, mean (SD), mg/dL	8.8 (2.9)	8.4 (3.0)	13.6
Antidepressant use	37 (44.6)	21 (27.3)	36.7
Opioid use	24 (28.9)	24 (31.2)	5.0
FACIT-F fatigue score	27.7 (10.6)	28.9 (11.4)	10.9
FACIT-F score ≤44	80 (96.4)	72 (93.5)	13.3
BPI average pain severity score	4.0 (3.3)	3.3 (3.2)	21.5
BPI score ≥5	43 (51.8)	31 (40.3)	23.2
BDI-II depression score	16.3 (9.1)	14.7 (7.9)	18.8
BDI-II score ≥16)	37 (44.6)	32 (41.6)	6.1

Abbreviations: BDI-II, Beck Depression Inventory II; BPI, Brief Pain Inventory Short Form; CCI, Charlson Comorbidity Index; ESKD, end-stage kidney disease; EDW, estimated dry weight; FACIT-F, the Functional Assessment of Chronic Illness Therapy Fatigue; IDWG, interdialytic weight gain percentage (of postdialysis weight during preceding 1 month).

### Adherence to Intervention

All the participants in the intervention group chose to receive CBT. Adherence to intervention was high; that is, 80% of patients in the intervention group and 95% of those in the control group completed at least 80% of the intended sessions (eTable 3 in Supplement 2). The average length of the sessions was 46 minutes in TĀCcare and 27 minutes in the control group. More than 95% of the total 1102 sessions in the study were completed during hemodialysis in the dialysis clinics, and the rest at home. Only 5 patients in the intervention group and 11 in the control group had medication initiation for pain and/or depression during the 3-month intervention period. Among those receiving antidepressants or opioid medications at baseline, the changes in average dosages during the 3-month period were small (eTable 4A and 4B in Supplement 2).

### Primary Outcome

**Figure 2.**
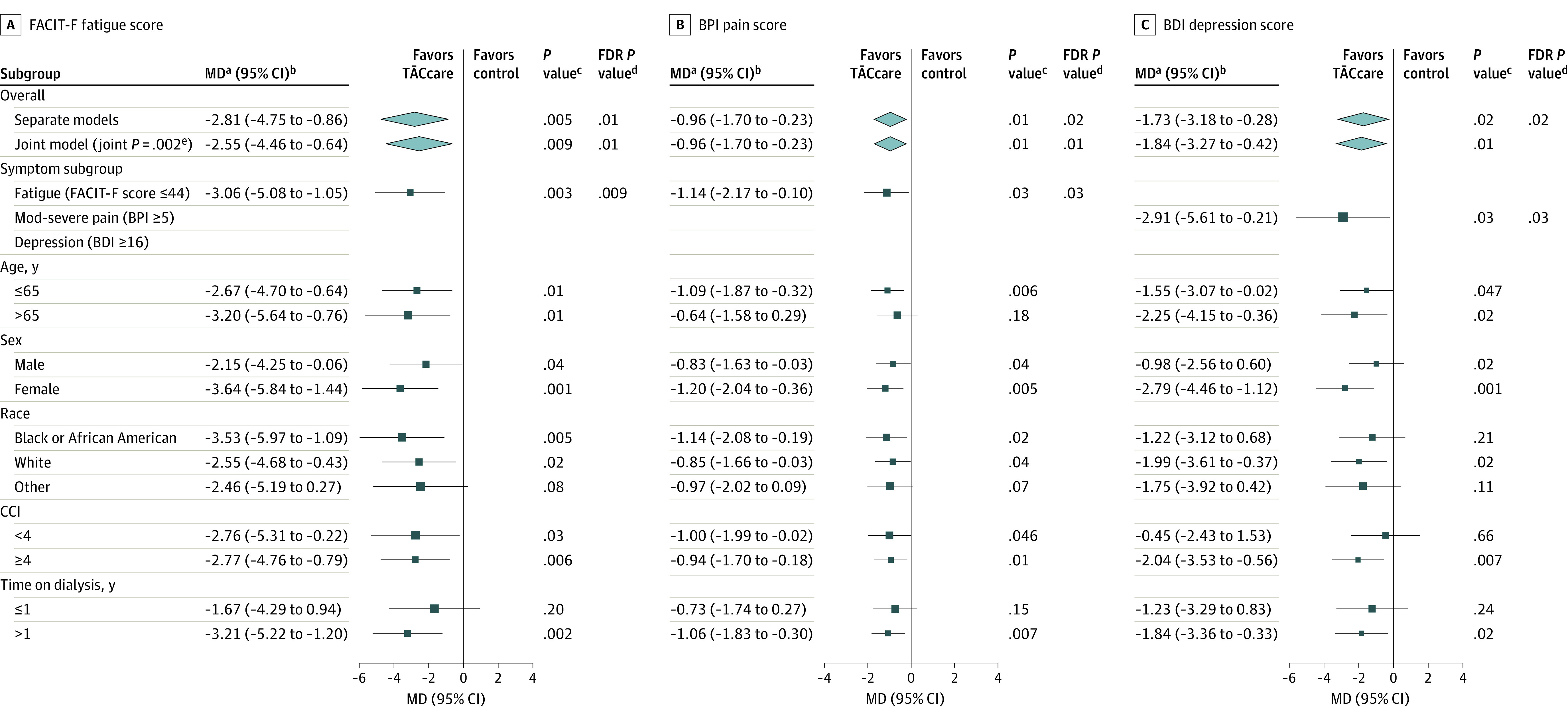
Primary and Subgroup Analysis of Effect of TĀCcare Intervention vs Control on Symptom Levels at 3 Months BDI-II refers to the Beck Depression Inventory II in which higher scores indicate more depression; BPI, Brief Pain Inventory Short Form, higher scores indicate more pain; and FACIT-F, Functional Assessment of Chronic Illness Therapy Fatigue in which higher scores indicate less fatigue. ^a^Adjusted mean difference (MD) from mixed model contrast; md = Δ control – Δ TĀCcare for FACIT-Fatigue; md = Δ TĀCcare – Δ control for BPI-pain and BDI-depression. ^b^95% CIs without multiplicity correction from mixed-model contrast. ^c^Raw *P* value from mixed-model contrast. ^d^Benjamini-Hochberg corrected *P* value for false discovery rate. ^e^Joint-test *P* value for the intervention effects on the 3 primary outcomes from the joint longitudinal model.

In the intention to treat analyses, compared with the control group, patients in the intervention group experienced significantly larger reductions in fatigue (mean difference [md], 2.81; 95% CI, 0.86 to 4.75; FDR *P* = .01), pain severity (md, −0.96; 95% CI, −1.70 to −0.23; FDR *P* = .02); and depression (md, −1.73; 95% CI, −3.18 to −0.28; FDR *P* = .02) at 3 months. Analyses from the joint model were very similar (fatigue: md, 2.55; 95% CI, 0.64 to 4.46; FDR *P* = .01; pain: md, −0.97; 95% CI, −1.70 to −0.23; FDR *P* = .01; depression: md, −1.84; 95% CI, −3.27 to −0.42; FDR *P* = .01) at 3 months. When restricted to those who had elevated levels of specific symptom(s) at baseline, there were more pronounced clinically significant improvements in fatigue and pain at 3 months (Figure 2 and Table 2). When restricted to those with depression at baseline, the participants in the intervention group experienced a 27.1% improvement in BDI-II scores at 3 months compared with baseline, which was greater than the clinically meaningful threshold of 17.5%.^48^ However, compared with the control group, the relative change was statistically significant but not clinically meaningful at 3 months (md, 13.3%). Subgroup analyses showed no significant difference in effect estimates among groups based on age, sex, race, CCI, and time on dialysis (years), except for a larger reduction in depression among women vs men (difference in md, −1.81; 95% CI −3.27 to −0.35; *P* = .02; Figure 2).

**Table 2.** Treatment Effectiveness End Points After 12 Weeks of TĀCcare Intervention vs Attention Control

Outcome	Change from baseline to wk 12, mean (95% CI)		Adjusted mean group difference	*P* value	
	TĀCcare (n = 83)	Control (n = 77)		Group difference	MCA
**Primary outcomes: intervention effects from separate mixed models**					
FACIT-F fatigue score (range 0-52; higher = less fatigue; CID 3-4)^41,42^	3.452 (2.014 to 4.891)	0.646 (−0.662 to 1.955)	2.806 (0.861 to 4.750)	.005	.014
BPI average pain severity score (range, 0-10; higher = worse pain; CID 1)^43,47^	−0.949 (−1.491 to −0.407)	0.016 (−0.487 to 0.518)	−0.965 (−1.704 to −0.226)	.011	.016
BDI-II depression score (range, 0-63; higher = worse depression; CID, 17.5% reduction from baseline)^48^	−2.934 (−4.007 to −1.860)	−1.203 (−2.178 to −0.228)	−1.731 (−3.181 to −0.281)	.019	.019
**Primary outcomes: intervention effects from joint longitudinal model (joint *P* = .002)**					
FACIT-F fatigue score	3.344 (1.937 to 4.750)	0.794 (−0.494 to 2.083)	2.549 (0.641 to 4.457)	.009	.011
BPI pain score	−0.944 (−1.479 to −0.408)	0.021 (−0.480 to 0.522)	−0.965 (−1.699 to −0.231)	.010	.011
BDI-II depression score	−2.986 (−4.039 to −1.932)	−1.143 (−2.106 to −0.181)	−1.842 (−3.270 to −0.415)	.011	.011
**Primary outcomes: restricted to only those experiencing the symptom at baseline** ^a^					
Fatigue (FACIT-F score ≤44)	3.731 (2.247 to 5.215)	0.668 (−0.694 to 2.031)	3.063 (1.048 to 5.078)	.003	NA
Pain (BPI score ≥5)	−2.248 (−2.976 to −1.519)	−1.112 (−1.850 to −0.374)	−1.135 (−2.172 to −0.099)	.032	NA
Depression (BDI-II score ≥16)	−5.687 (−7.691 to −3.684)	−2.777 (−4.587 to −0.967)	−2.910 (−5.610 to −0.210)	.035	NA
**Secondary patient-reported outcomes at 3 mo**					
BPI-7 interference (range 0-10; higher = worse interference)	−0.757 (−1.303 to −0.211)	−0.024 (−0.528 to 0.480)	−0.734 (−1.477 to 0.010)	.05	NA
BPI-4 severity (range 0-10; higher = worse severity)	−0.802 (−1.322 to −0.282)	0.095 (−0.386 to 0.577)	−0.898 (−1.606 to −0.189)	.013	NA
PCS (range 0-100; higher = better physical health)	1.311 (0.142 to 2.480)	0.432 (−0.633 to 1.496)	0.879 (−0.702 to 2.461)	.28	NA
MCS (range 0-100; higher = better mental health)	1.535 (0.176 to 2.895)	0.828 (−0.428 to 2.085)	0.707 (−1.145 to 2.559)	.45	NA
GAD anxiety score (range 0-21; higher = worse anxiety)	−1.336 (−1.982 to −0.690)	−0.825 (−1.414 to −0.236)	−0.511 (−1.386 to 0.364)	.25	NA
Perceived social support (range, 12-84; higher = more support)	−0.014 (−1.701 to 1.673)	0.697 (−0.851 to 2.245)	−0.711 (−3.001 to 1.579)	.54	NA
PASE physical activity (range, 0-400; higher = greater activity)	−3.664 (−12.739 to 5.411)	0.052 (−8.423 to 8.526)	−3.716 (−16.138 to 8.706)	.56	NA
PROMIS fatigue (T score range, 0-100; higher = more fatigue)	−3.163 (−4.463 to −1.862)	−1.571 (−2.748 to −0.394)	−1.592 (−3.346 to 0.163)	.08	NA
PROMIS depression (T range 0-100, higher = worse depression)	−2.889 (−4.212 to −1.566)	−0.350 (−1.564 to 0.865)	−2.539 (−4.336 to −0.743)	.006	NA
PROMIS pain interference (T score range, 0-100; higher = worse pain)	−1.157 (−2.479 to 0.166)	−0.249 (−1.477 to 0.980)	−0.908 (−2.713 to 0.898)	.32	NA
PSQI sleep (range 0-21; higher = worse sleep)	−0.821 (−1.331 to −0.311)	−0.514 (−0.974 to −0.054)	−0.308 (−0.994 to 0.379)	.38	NA
**Secondary adherence outcomes at 3 mo**					
Medication/dietary adherence^b^	−0.001 (−0.104 to 0.102)	−0.088 (−0.197 to 0.020)	0.087 (−0.062 to 0.237)	.25	NA
Fluid restriction adherence (IDWG ≤ 3.5%)	−0.059 (−0.131 to 0.013)	−0.037 (−0.099 to 0.025)	−0.022 (−0.117 to 0.073)	.65	NA
HD treatments missed in past 30 d, No.	−0.012 (−0.238 to 0.215)	−0.103 (−0.300 to 0.095)	0.091 (−0.209 to 0.392)	.55	NA

Abbreviations: BPI, Brief Pain Inventory Short Form; BPI-4, pain severity score (average of the 4 pain severity items); BPI-7, pain interference score (average of 7 pain interference items); BDI-II, Beck Depression Inventory II; CID, clinically important difference; GAD, Generalized Anxiety Disorder-7; HD, hemodialysis; IDWG, interdialytic weight gain percentage of postdialysis weight during preceding 1 month; MOS, Medical Outcomes Study; MCA, multiple comparison adjusted; MCS, Short Form-12 Mental Component Score; PASE, Physical Activity Scale for Elderly Short Form-12; PCS, Short Form-12 Physical Component; PROMIS, National Institutes of Health Patient Reported Outcomes Measurement Information System Adult Global Health; PSQI, Pittsburgh Sleep Quality Index.

^a^ Number with clinically meaningful symptoms overall and within each group, as shown in Table 1.

^b^ Adherence defined as serum phosphorus level <6.0 mg/dL or >1.0 mg/dL decline in serum phosphorus from preceding month when absolute serum phosphorus level is >6.0 mg/dL.

### Secondary Outcomes

**Figure 3.**
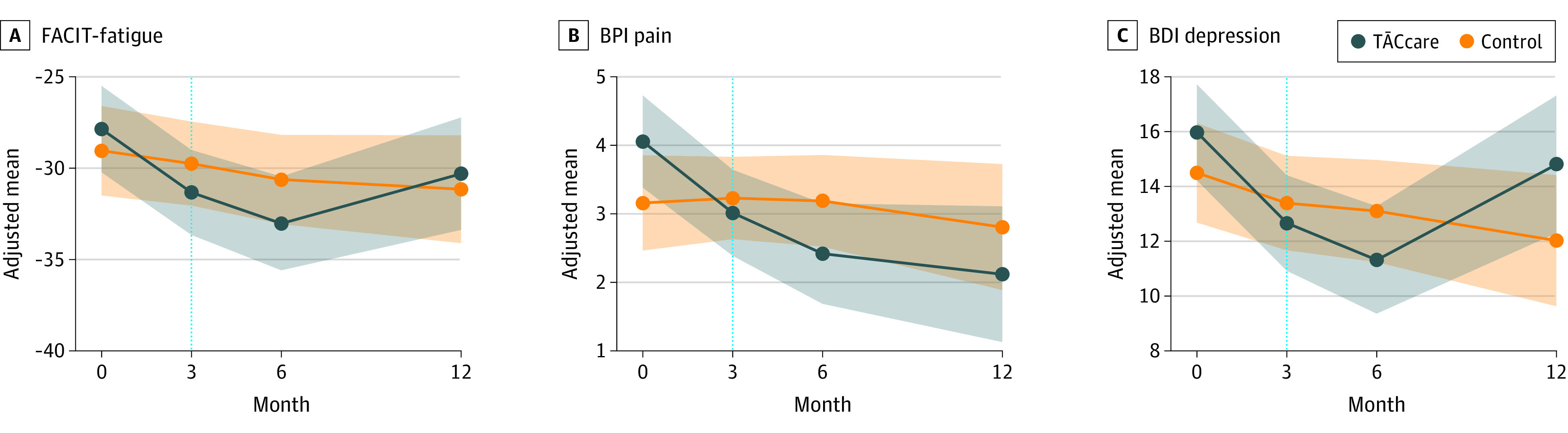
Adjusted Mean Effects of TĀCcare Intervention vs Control on Symptom Levels at 3-, 6- and 12-Month Time Points Adjusted mean effects of TĀCcare intervention vs control on symptom levels at 3-, 6- and 12-month time points. BDI refers to the Beck Depression Inventory-II in which higher scores indicate more depression; BPI, Brief Pain Inventory Short Form, higher scores indicate more pain; and FACIT-F refers to the Functional Assessment of Chronic Illness Therapy Fatigue in which the original score was multiplied by −1 so that higher scores indicated more fatigue.

At 6 months, significant improvements in fatigue and pain were sustained (FACIT-F md, 3.73; 95% CI, 0.87 to 6.60; *P* = .01; BPI md, −1.49; 95% CI, −2.58 to −0.40; *P* = .008), but there was no significant improvement in depression (md BDI, −2.18; 95% CI, −4.36 to 0.01; *P* = .05). There were no significant effects of intervention on fatigue, pain and depression at 12 months as compared to controls (Figure 3). The PROMIS depression scores improved significantly at 3 months (md, −2.54; 95% CI, −4.34 to −0.74; *P* = .006) and 6 months (md, −3.45; 95% CI, −6.06 to −0.84; *P* = .01). There were no significant treatment effects on HRQOL, pain interference, sleep quality, anxiety, physical activity, perceived social support, or adherence outcomes at any time point (Table 2). There were no significant differences in adverse events among the treatment groups. The only study-related adverse event was a 1-time infiltration of vascular access while using the iPad during hemodialysis (eTable 5 in Supplement 2). Among the 19 patients who completed the satisfaction survey, more than 80% felt the program was beneficial and would enroll in it again.

## Discussion

In this multicenter randomized clinical trial targeting key symptoms in patients receiving long-term hemodialysis, a stepped collaborative care intervention led to clinically significant improvements in fatigue and pain at 3 months, which were sustained for 6 months. Improvements in depression at 3 months were statistically significant but small. Targeting symptom clusters allowed for improvement in multiple symptoms that commonly coexist. When presented with therapeutic options, most patients preferred CBT over pharmacotherapy; adherence to CBT was high. Lastly, telemedicine-delivered collaborative care was effective, safe, and reliable during in-center hemodialysis.

The TĀCcare study addresses gaps in the care for this population given that previous work has shown a marked symptom burden that is underrecognized and poorly treated.^6,7,49^ This is especially true for fatigue and pain, the 2 most prevalent symptoms for which treatment options have been limited to medications and exercise, with studies showing limited efficacy and mixed results.^15,17,50,51^ To our knowledge, evidence on fatigue- or pain-specific psychosocial interventions among this population has been very limited and based on small single-center studies with methodologic limitations.^15,16,17^ Our trial provides rigorous data from a large multicenter cohort of diverse backgrounds on effectiveness of a collaborative care intervention in this population. This study also addresses symptoms that are highly prioritized for treatment by patients and key stakeholders.

Our collaborative care intervention extends findings from other populations with chronic illness, such as patients with heart failure and cancer.^52,53^ This approach provides an integrated multidisciplinary management plan that is individualized according to each patient’s clinical status, preferences, and treatment response. We observed an approximately 6% improvement in energy level and a 10% improvement in pain severity with TĀCcare intervention vs the control health education. These improvements were within the range of clinically important differences reported in cancer and other disease states, ie, 3 to 4 for FACIT-F score and 1 for BPI.^29,42,43,47,54^ For both fatigue and pain, the treatment effects were sustained for 6 months but were not sustained at the 12-month follow-up, which suggests that follow-up booster CBT sessions may be needed for long-term symptom improvement.

In the present study, improvements in depression were statistically significant but small. This finding contrasted with those of prior studies which showed significant improvements with CBT in patients undergoing hemodialysis.^11,12^ This difference may be associated with the lower severity of depression at baseline in our study. The ASCEND trial showed improvement in depression with CBT or sertraline at 12 weeks among 120 randomized patients.^12^ However, the chairside CBT used by that study would likely be impractical to implement in a clinical setting given the lack of trained therapists willing to travel to dialysis units and the logistical challenges with scheduling chairside CBT. By using technology to deliver CBT, the TĀCcare intervention can overcome many of these and other patient-related barriers, such as scheduling face-to-face appointments, transportation, and access to trained therapists. Technology assisted care provides a resource efficient and scalable strategy that can be readily adopted and widely disseminated in routine dialysis care.

Unlike some of the prior trials in long-term hemodialysis,^12,50^ TĀCcare offered a choice of both CBT and pharmacotherapy, thus allowing for incorporation of patient preferences and tailoring of treatment plan. Interestingly, only 5 patients in TĀCcare intervention group requested medication (for pain or depression), and all patients participated in CBT. Prior studies on symptom management have shown patient reluctance to receive pharmacotherapy owing to the additional pill burden and/or adverse effects from medication interactions.^50,55,56^ By offering treatment options and involving patients in shared decision-making, TĀCcare provided an individualized approach that allowed patients to choose which treatment strategies they received, which yielded high acceptance and adherence.

### Strengths and Limitations

A strength of this study was the diverse racial and ethnic representation from 2 geographic areas, which enhances its external validity. In addition, the trial included urban and rural dialysis units, which provided evidence for use of this collaborative care model to address symptom burden among underserved populations. The adherence rate to CBT was high, and outcomes were assessed centrally by trained interviewers who were blinded to treatment assignment. Lastly, the use of an attention control instead of usual care reduced bias and enhanced interpretability of the results.

Study limitations included higher than expected loss of follow-up owing in part to changes in practice in response to the COVID-19 pandemic. This loss was addressed by increasing recruitment goals to offset the observed dropouts. Screening tests for fatigue and pain used single-item assessments that have not been validated and may have missed some true positives. This trial had a limited ability to test for heterogeneity of treatment effects. Lastly, there may be future opportunities to culturally tailor the intervention.

## Conclusions

The TĀCcare trial is the first randomized clinical trial, to our knowledge, for people with kidney disease where the intervention is targeted toward multiple symptoms rather than a single symptom. Our results showed that among patients undergoing hemodialysis, a 12-week CBT-based stepped collaborative care intervention can offer clinically significant improvements in fatigue and pain. Leveraging telemedicine to deliver CBT that targets symptom clusters during hemodialysis sessions may provide a scalable and resource-efficient approach to improve patient-centered outcomes among patients with ESKD who are undergoing long-term hemodialysis.

## Notes

Supplement 1.
Trial Protocol

Supplement 2. **eMethods**. Statistical Analysis Plan **eFigure.** Distribution of patient symptoms at baseline **eTable 1.** Symptom scores using validated questionnaires at baseline among those with fatigue, pain or depression at screening **eTable 2.** Baseline levels of other patient-reported outcomes and adherence to medications, diet, fluid restriction and hemodialysis treatments **eTable 3.** Summary of telemedicine sessions for TĀCcare study participants **eTable 4a.** Change in proportion of patients reporting using antidepressant or analgesic medications from baseline to 3-months **eTable 4b.** Change in average medication dose from baseline to 3-months among participants reporting use at baseline **eTable 5.** Adverse events by TĀCcare study group

Supplement 3.
Data Sharing Statement
