# Supplementary material for: Effects of Technology Assisted Stepped Collaborative Care Intervention to Improve Symptoms in Patients Undergoing Hemodialysis: The TĀCcare Randomized Clinical Trial
Source: JAMA Intern Med. 2023 Jun 20;183(8):795–805. doi: 10.1001/jamainternmed.2023.2215 (PMC10282960; doi:10.1001/jamainternmed.2023.2215)
Supplement: Supplement 1. — Trial Protocol [file jamainternmed-e232215-s001.pdf]

Rationale and design of Technology Assisted stepped Collaborative Care Intervention to Improve Patient-centered Outcomes in Hemodialysis Patients (TĀCcare trial)

Study Protocol

Protocol Synopsis

**Protocol Title/number**	**T**echnology **A**ssisted stepped **C**ollaborative **Care** Intervention to Improve Patient-centered Outcomes in Hemodialysis Patients (TĀCcare)
**Grant Number**	R01DK114085-01
**Brief Statement of Purpose of Study**	This clinical trial will compare the effectiveness of a 12-week stepped collaborative care intervention with an attention control arm of technology-delivered health education. Hemodialysis (HD) patients will be recruited and will be evaluated for key patient-centered outcomes post 12 weeks intervention, and at 6 months and 12 months.
**Principal Investigator**	Manisha Jhamb, MD MPH
**Co-PIs, Lead Study Coordinator, Statistician, Coordinating Center PI (if applicable)**	Mark Unruh, MD, MS, Site PI<br>Scott Beach, PhD, Co-Investigator<br>Marilena Roumelioti, MD, Co-Investigator<br>Bruce Rollman, MD, Co-Investigator<br>Sarah Erickson, PhD, Co-Investigator<br>Jennifer Steel, PhD, Co-Investigator<br>Yoram Vodovotz, PhD, Co-Investigator<br>Steve Weisbord MD, Co-Investigator<br>Jonathan Yabes, PhD, Biostatistician<br>Melissa Weimer, MS, Project Coordinator
**List of Participating Clinics, Data Centers, Resource Centers**	2 Clinical Sites:<br>University of Pittsburgh<br><br>University of New Mexico
**Study Commencement Date**	July 01, 2017
**Target Enrollment**	*160 (revised as of 5/2020; original Target 150)*
**Study Design**	Parallel arm randomized controlled trial
**Study Objectives/Aims**	**Aim 1:** To test the effectiveness of a 12-week TĀCcare to reduce depression, pain and fatigue and improve HRQOL in HD patients as compared to health education control<br><br><u>Primary hypotheses:</u> Patients randomized to TĀCcare will report improvement in our primary outcome of clinically meaningful change in depression, pain or fatigue as ascertained blindly by the BDI-II, FACIT-F and BPI questionnaires respectively, after a 12-week intervention as compared to control arm. TĀCcare will also improve HRQOL and EMA-measured composite score of fatigue, sleepiness and mood (secondary outcomes) after a 12-week intervention as compared to the controls. We will monitor maintenance of changes of these symptoms for 12 months.<br><br>**Aim 2:** To determine the effect of TĀCcare on adherence to medications, diet and hemodialysis treatments.

	Secondary hypotheses: Patients randomized to TASCCI will have at least a 25% improvement in adherence to medications, diet and HD treatments after a 12 week intervention as compared to the control arm.<br><br>**Aim 3:** To explore effect of TĀCcare on biomarkers of inflammation<br><br>Secondary hypotheses: Patients randomized to the 12 week TASCCI will have clinically meaningful improvements in inflammatory cytokines (*i.e., hsCRP, IL-6, IL-10,TNF-α*) as compared to the controls.
**Study Intervention**	Technology Assisted stepped Collaborative Care (TĀCcare)<br>Technology Assisted Health Education Attention Control
**Intervention Duration (per participant)**	TĀCcare: 12 weekly sessions<br>Control: 6 sessions over 12 weeks
**Follow-up Duration (per participant)**	12 months
**Inclusion Criteria**	1. 18 years or older<br>2. undergoing thrice-weekly maintenance HD for over 3 months<br>3. English speaking<br>4. able to provide informed signed consent<br>5. no evidence of thought disorder, delusions, or active suicidal intent is observed or reported
**Exclusion Criteria**	1. evidence of thought disorder, delusions or active suicidal intent<br>2. active substance abuse<br>3. too ill or cognitively impaired to participate based on clinicians' judgement<br>4. anticipated life expectancy of less than 1 year<br>5. unable or unwilling to adhere to study protocol<br>6. participating in another clinical trial or taking an investigational drug<br>7. scheduled for living donor kidney transplant within the next 6 months<br>8. relocating to another dialysis unit within 6 months<br>9. transitioning to home dialysis over next 6 months
**Screening Criteria** {patients screened after initial eligibility screening based on inclusion/exclusion criteria}	1. symptomatic with clinical levels of at least one of the 3 symptoms (≥10 on PHQ-9 and/or ≥4 on pain scale and/or ≥5 on fatigue scale)<br>2. At least in contemplation stage of behavior change
**Study Outcomes**	The **primary outcomes** will be depression, pain or fatigue as ascertained blindly by the BDI-II, FACIT-F and BPI questionnaires respectively, at 12-weeks. Secondary outcomes include HRQOL and EMA-measured composite score of fatigue, sleepiness and mood; adherence to medications, diet and hemodialysis treatments; and inflammatory cytokines (i.e., hsCRP, IL-6, IL-10,TNF-α)
**Study Stopping Rules**	No statistics-based stopping rules have been established.

Introduction

Most hemodialysis (HD) patients experience symptoms of depression, pain and fatigue that impair their health-related quality of life (HRQOL) significantly. These symptoms are associated with increased hospitalization and mortality, mediated by behavioral factors (e.g. non-adherence to medication and dialysis) and biological factors (e.g. inflammatory cytokines). Prior interventions to alleviate symptoms and improve HRQOL showed limited effectiveness in HD patients and their effect on bio-behavioral mediators is lacking evidence. It is imperative to improve patient-centered dialysis care and to address call from Kidney Disease

Improving Global Outcomes (KDIGO) guidelines for integration of symptom assessment and management in routine HD-care. **T**echnology-**A**ssisted stepped **C**ollaborative **C**are (TĀCcare) is a multi-center randomized controlled trial (RCT) of 150 diverse HD patients from Pennsylvania and New Mexico, designed to compare the effectiveness of a 12-week stepped collaborative care intervention (cognitive behavioral therapy, CBT) with an attention control arm of technology-delivered health education. Collaborative care provides an integrated multi-disciplinary structured management plan. Furthermore, a stepped approach to pharmacotherapy and/or CBT allows for individualization of treatment according to patients' clinical status, preferences and treatment response. To simplify the delivery of CBT and to minimize patient and provider burden, we will use live video-conferencing with patients in dialysis units. We will examine the effect of these interventions on patient symptoms, HRQOL, treatment adherence and inflammatory biomarkers. This RCT tests a readily implementable intervention that can be integrated in routine HD-care and will generate novel and meaningful insights on strategies to alleviate common symptoms and improve HRQOL in HD.

Background

Patients with End-Stage Renal Disease (ESRD) on hemodialysis (HD) experience a symptom burden comparable to advanced cancer patients.[1-4] Fatigue, pain and depression are among the most debilitating symptoms, reported by more than 60%, 50% and 20% of HD patients, respectively.[5-8] These symptoms often co-exist as "symptom-clusters," can exacerbate one another, and are independently associated with poor health-related quality of life (HRQOL) in HD.[2,9-12] Given their associations with decrements in multiple HRQOL domains, these symptoms are associated with medication and HD sessions non-adherence. [13-15] Non-adherence, as well as biological factors (e.g. inflammatory cytokines), likely contribute to increased hospitalization and mortality in these patients.[9,15-18] Additionally to survival, improvement of these symptoms is extremely important to HD patients and their caregivers.[19,20] Only recently, the Kidney Disease Improving Global Outcomes (KDIGO) Controversies Conference on Supportive Care (2015) advocated for integration of symptom assessment and management into routine ESRD care. [21] Additionally, recent Center for Medicare and Medicaid Services (CMS) criteria for ESRD Quality Incentive Program mandate reporting of depression screening and treatment for all patients.

Unfortunately, routine treatment approaches for symptoms in HD patients had limited success.[5,7,8] Although promising, studies on the efficacy of pharmacotherapy or psychotherapy [such as cognitive behavioral therapy (CBT)] for depression in HD are methodologically limited (small sample size, observational design, lack of adequate control group, lack of adequate minority racial/ethnic representation).[23-28] Data also suggest benefits of analgesic medications for pain; and of sleep hygiene, exercise and anemia correction for fatigue.[7,29] No studies have tested the efficacy of CBT for pain or fatigue in ESRD. However, CBT has proven efficacious in similarly symptomatic cancer patients and is based on a conceptual model that highlights the importance of precipitating and perpetuating factors (e.g. poor sleep, physical inactivity, and coping strategies) which exist in ESRD patients.[30,31] Key reasons for poor acceptance and suboptimal results of prior interventions were: failure to target "symptom-clusters", combining pharmacotherapy and behavioral-therapy, incorporating patient treatment preferences, or integrating interventions into existing dialysis-care team-models.[8,32,33]

Rationale for Study

The TĀCcare trial is based on a Stepped-Collaborative Care Intervention (SCCI) that addresses these barriers to effective treatment implementation and presents an ideal symptom management strategy for HD patients. SCCI emphasizes a more real-world and flexible intervention that has multiple "active ingredients" (psychotherapy and pharmacotherapy) and includes a care manager actively monitoring treatment response, following an evidence-based protocol to modify treatment content and intensity, and serving as a liaison between the health-care teams to facilitate integration of mental and physical-health treatments.[34,35] In SCCI, patients' adherence, treatment response, preferences and outcomes are monitored actively and treatments are modified as needed to achieve the best possible outcome for each patient. To simplify the delivery of the behavioral therapy component and reduce the patient and provider burden, the current trial will incorporate video-conferencing in dialysis units. This will be the first, multi-center randomized controlled trial (RCT) targeting symptom-clusters and using a SCCI with technology-based delivery of behavioral interventions in HD patients. The results will lead to new approaches for symptom management in HD patients.

Methods

Study Overview: This study is a two-site, parallel group, RCT comparing a technology assisted SCCI (TĀCcare) to technology-assisted health education control in improving key patient-centered outcomes in 150 HD patients. After signing informed consent, completing baseline assessments and meeting eligibility criteria, enrolled patients will be randomized 1:1 to TĀCcare or technology-delivered health education and will undergo a 12-week intervention. The study participants will be recruited using an approach to limit drop-out while enhancing generalizability and the main analysis is intention-to-treat. The main patient-centered outcomes **(Aim 1)** are: 1) a clinically meaningful change (effect size 0.6) in depression, pain or fatigue *(primary outcome)*; 2) a change in HRQOL and ecological momentarily assessed composite symptom score of fatigue, sleepiness and mood *(secondary outcomes)* after the 12-week intervention. We will also determine the effect of TĀCcare on adherence to medications, diet and HD treatments *(secondary outcomes)* **(Aim 2)** and we will explore changes in inflammatory biomarker levels **(Aim 3)** after the 12-week intervention. Since the effect of pharmacotherapy may be delayed, we will measure these outcomes at 6 months and again at 12 months to assess the maintenance of the benefits of our intervention.

Setting and Participants: A total of 150 adult chronic HD patients will be recruited from the two clinical sites at the University of Pittsburgh and the University of New Mexico which collectively have 20 free-standing dialysis clinics that vary in geographic areas, size and population served. These clinics were selected to ensure a diverse HD population with adequate representation of females and minority groups including African Americans, American Indians and Hispanic/Latinos.

Eligibility Criteria:
Patient inclusion criteria include:
a) 18 years or older
b) undergoing thrice-weekly maintenance HD for over 3 months
c) English speaking
d) ability to provide informed signed consent
e) no evidence of thought disorder, delusions, or active suicidal intent observed or reported.

Patient exclusion criteria include:
a) evidence of thought disorder, delusions or active suicidal intent – observed or reported
b) active substance abuse
c) too ill or cognitively impaired to participate based on clinicians' judgement
d) anticipated life expectancy of less than 1 year
e) unable or unwilling to adhere to study protocol
f) participating in another clinical trial or taking an investigational drug
g) scheduled for living donor kidney transplant within the next 6 months
h) relocating to another dialysis unit within 6 months.

We will not exclude patients who are already being treated for pain or depression if they screen positive for clinical levels of depression, pain or fatigue as studies suggest that these symptoms are often undertreated in HD patients. [36,37]

Recruitment and Consent procedures: The research team will work with dialysis clinic staff (charge nurse, social worker) to identify patients who are suitable to be approached for the study and introduce the research team to patients who are interested in participating in the study. They will also provide informational material for patients who may be interested in learning more about the study by contacting a member of the research team. The research team will assess the patient's eligibility for the study. Interested patients will be approached by the research team during their regularly scheduled HD treatment to explain the study purpose and procedures. We will adhere to dialysis unit policies to ensure privacy and social distancing while doing this, and this will be conducted either on the treatment floor or the waiting room (based on guidelines for research staff for the dialysis unit). Risks and benefits of the study will be explained and patients will be provided time to read the consent and ask questions. Interested patients will undergo brief screening and only those who screen positive for clinical levels of one or more symptoms and are at least at the contemplation stage of behavior change will be enrolled in the study after informed consent is obtained.

Screening: Eligible patients will be screened for: 1) Clinical levels of symptoms: Screening will include 9 questions for depressive symptoms from the Patient Health Questionnaire-9 (PHQ-9), 0-10 point Likert scale of

pain (10=worst pain), and 0-10 point Likert scale of fatigue (10=worst fatigue) experienced within last 2 weeks.[38] Only symptomatic patients who have clinical levels of at least one of the 3 symptoms (≥10 on PHQ-9 and/or ≥4 on pain scale and/or ≥5 on fatigue scale) will be included in the study. This is important because we are targeting patients with high symptom burden to evaluate the efficacy of TĀCcare.
2) Readiness for Behavior Change: We will use a 5–item Stages of Behavior Change questionnaire to assess patient's readiness for treatment. A clinically significant effect size has been shown for the association between stage of change and psychotherapy outcomes, suggesting that patients who are in the pre-contemplation stage with no intention to change behavior in the foreseeable future are unlikely to actively participate and derive benefit from the treatment.[39] Thus, we will enroll only those patients who are at least in contemplation stage of behavioral change for symptom management ("would be motivated to seek treatment or talk to their doctor about it").

Randomization: After completion of baseline assessments (as below), the Study Coordinating Center will randomly assign patients in 1:1 ratio to either TASCCI or health education arm, using a pseudo-random number generator. Randomization will be stratified by the two clinical sites, age (< 60 and ≥ 60 years) and Charlson comorbidity index (<4 and ≥4) to achieve equal distribution of these potentially confounding variables between the two arms. Randomly-permuted blocks will be used to help balance numbers of participants assigned to each intervention. Patients in both arms will receive an intervention delivered using video-conferencing. Thus, even though patients at outpatient dialysis clinics are in close proximity to fellow patients, we expect that any chances of cross-contamination between study arms is minimal. Moreover, even if the patients in different arms at the same unit interact with each other, these interactions are likely to be brief and unlikely to have any sustained effect on our outcomes of interest. Moreover, the care coordinator for the intervention and control arm will not be the same person to ensure there is no unintentional contamination from the study staff while delivering the intervention. Lastly, although there is a possibility that the renal providers start recognizing and recommending symptom management in the control arm patients, this is unlikely given reluctance of patients and providers regarding pharmacotherapy alone, and patient burden associated with in-office CBT.
Allocation concealment and blinding: Neither the patients nor the PI can be blinded due to the nature of the study intervention. However, research staff assessing patient-centered outcomes will be blinded to the group assignment.

Treatment Intervention Study Arms
A) Technology Assisted Stepped Collaborative Care Intervention (TĀCcare): Patients randomized to the intervention arm will receive a stepped-care approach of pharmacotherapy and/or CBT for a 12-week period. The intervention will target one or more symptoms based on patients' report of clinical levels of each symptom and patient preference. A local care coordinator at each clinical site will be assigned to each patient. During weekly online video-conferencing sessions, care coordinators will engage the patient actively, encourage and monitor treatment adherence, and assess response and side-effects. Both clinical sites have secure and established telemedicine portals that are routinely used for clinical care and research (Vidyo at University of Pittsburgh and Echo Zoom at University of New Mexico). These live video-based face-to-face sessions will be conducted during one of the thrice weekly HD sessions at the dialysis unit with optional privacy screen. For each dialysis unit, the study will provide iPads, portable secure Wi-fi hotspot, wireless headphones and microphones. Similar to our pilot study (manuscript in preparation), these devices will be pre-programmed to ensure patient's ease and independence of use with minimal training, even among the elderly or those with limited technology experience, thus causing minimal additional burden for the dialysis staff. If there are any medical emergencies during the HD session, the video-based session will be cancelled and rescheduled once the patient is clinically stable. If the patients have the needed technology at home, they will have the option of doing these video-conferencing sessions at home.

B) Stepped Approach to Individualize Treatment: Treatment options include pharmacotherapy or CBT or both, based on patient's symptom severity and preference. As the causes for fatigue in these patients are multifactorial[7,40], fatigue management algorithm will include assessment of depressive symptoms, sleep quality, pain, physical inactivity and medication side-effects and these contributing factors will be addressed as needed. In addition, we will ensure that dialysis dose and hemoglobin are in target ranges.

At the first weekly video-conferencing session, the care coordinator will identify which symptom/symptoms the patient would like to address, explain the risk and benefits of treatment options in a way that is tailored to the patient's health literacy level, stress the voluntary nature of their treatment preferences, and choose a treatment plan based on shared decision-making. Subsequently, the care coordinators will conduct weekly 45 to 60-minute video-conferencing sessions over 12 weeks and will tailor behavioral therapy sessions and/or recommend pharmacotherapy based on an evidence-based protocol. Principles for measurement-based care will be followed every 2 weeks, i.e. changes to treatment will be based on assessment of response to treatment, severity of symptoms and side effects of treatment.[41] Treatment response and patient preferences will inform care coordinator's decision to modify the treatment for which they will follow an evidence-based protocol to add/intensify psychotherapy and/or pharmacotherapy.

C) Psychotherapy/ Cognitive Behavioral Therapy: A short-term focused psychotherapy approach called CBT will be employed to reduce the symptoms of depression, pain and fatigue and will be individualized based on patient's symptom severity, preferences and treatment response. This American Psychiatric Association recommended approach helps patients learn self-help skills to deal with real-life issues. CBT has been shown to be highly efficacious with a moderate effect size in the management of these symptoms.[43] CBT strategies were adapted for HD patients to address challenges and needs in the context of ESRD and HD-dependence and were tested in our pilot study (manuscript under preparation). Specific CBT modifications included: (1) behavioral/psycho-education emphasizing the potential contribution of the diagnosis and treatment of ESRD that contributes to the symptoms burden; (2) identification of negative thought patterns regarding these symptoms and cognitive restructuring; (3) developing pleasant activities and strategies while considering the limitations of individuals with ESRD on HD; (4) identifying and resolving communication difficulties that may exist between the patient and his/her family or medical team that prevents management of symptoms; (5) relaxation and/or guided imagery; and (6) facilitating the understanding of how core beliefs and assumptions, that may be associated with ESRD and/or dialysis, may prevent effective symptom management.

D) Pharmacotherapy: The research team will follow an evidence-based protocol to recommend whether a medication should be initiated, changed or intensified. For pain management we will use the World Health Organization 3-step analgesic ladder modified to adjust medication dosage for HD. Such an approach has also been developed by the Mid-Atlantic Renal and Kidney End-of-Life Coalitions through support by the Center of Medicare and Medicaid Services.[44] This approach has been successfully used in HD patients and has been highly efficacious in controlling pain in over 95% of participants.[29] Pain medication algorithms will be based on whether the patient has neuropathic pain, nociceptive pain or both. Pharmacological treatment of depression will be based on efficacy and safety evidence in HD patients and dose adjustment for HD will be recommended as appropriate. [33,45,46] The research team including nephrologists and internist with symptom management expertise, along with clinical psychologist will make medication recommendations. These providers have training and experience with pharmacotherapy for symptom management. The recommendations will be conveyed to the patients' primary care provider (PCP) and/or nephrologist by the care coordinator, who will also monitor anti-depressant and analgesic medication adherence and side effects during weekly sessions. . Once the patient's PCP and/or nephrologist approve of the medication change, the care coordinator will facilitate implementation of the treatment by communicating and coordinating with the dialysis team and pharmacy if needed, so as to minimize burden on dialysis staff. The research team will not prescribe or provide any medications. To ensure that patient's PCP and/or nephrologist prescribe the recommended by the study medications, we will conduct regular educational and outreach meetings, and will communicate regularly with the physicians throughout the study period.

E) Collaborative Care: An important aspect of TĀCcare is the integration of care provided as part of research with the patient's routine clinical care. The care coordinators will serve as a liaison between the patient and other health care professionals within the patients' medical team. This is important for providing a multi-disciplinary approach to the patient's physical and mental health, for aligning symptom management with the patient's overall plan of care, and for ensuring patient acceptance and adherence to the study team's recommendations. To facilitate such collaboration, the care coordinator will communicate (via email or phone or personal meeting) with the dialysis team at least monthly and whenever medication changes are recommended; participate in monthly dialysis care plan meetings whenever possible, and will discuss any pertinent issues with the dialysis care team (nephrologist, nurses or social worker) at least monthly. We will record the type and frequency of communication with the care team."

The care coordinator will also participate in clinical dialysis care plan meetings and will provide recommendations as appropriate. The care coordinators are Master's level behavioral therapists who received specific training in cultural competence on HD-specific issues and will work closely under the supervision of a local study psychologist.

Technology Assisted Health Education Attention Control: Patients randomized to this study group will be provided with iPads during HD (same as the intervention group) and will have weekly sessions to match the frequency of contact in the intervention group. To match the attention and expectations of patients in this group, a care coordinator will use video-conferencing to deliver health education. Although weekly sessions with care coordinator would have been ideal, this is not financially feasible. Thus, these sessions will alternate with sessions in which access to educational videos and online educational material from the National Kidney Foundation (NKF; www.nkf.org) will be provided. Topics relevant to ESRD such as transplantation, heart health, immunizations and age appropriate cancer screening, etc. will be covered.

Rationale for Attention Control in Study Design: It is essential to compare TĀCcare to a control group that receives a treatment that approximates the amount of time and attention received by the treatment group but is unlikely to have a specific effect on the study outcomes. This approach examines the rival hypothesis that improvements in the dependent variable occur because of participant expectancy and the attention received during the course of the treatment rather than from the treatment itself (the so-called Hawthorne effect).[47] The attention control intervention must be feasible and relevant to the needs and health of the participants and should also be designed to engage participants and to avoid dropouts or selection biases based on the requirements of the experimental intervention.[48] These concerns have factored into our selection of a health education attention control for this study since it is a key issue in patients undergoing maintenance HD, yet it is unlikely to influence our primary outcomes. Comparison to an attention control group will reduce the variability of factors and biases that might influence the outcomes, enhance the interpretability of the study results and enable evaluation of the true effects of our intervention.

Intervention Fidelity and Monitoring: To maintain internal validity and enhance intervention fidelity (e.g., consistency across care coordinators and adherence to treatment protocol), care coordinators will follow a study manual specific for the study arm and will receive standardized training in dialysis-specific CBT or health education. Fidelity will be assessed by reviewing 10% of randomly selected audiotaped video-conferencing sessions, and fidelity checklist, and feedback will be provided to the study staff.

Duration of Intervention and Follow-Up: Each participant will be treated in the assigned group for 12 weeks, death, or loss to follow-up (withdrawal from study, transfer of dialysis clinic, change to peritoneal dialysis or transplantation). The length of intervention was selected based upon national guidelines recommending 8-12 weeks of weekly CBT or initial pharmacological treatment of depression [50] as well as our prior results showing successful efficacy of a similar 12-week intervention in cancer patients. [51] If there is any acute change in patient's medical condition/hospitalization during the intervention period, patients will be re-evaluated for their ability and interest to continue participation once medically stable. If needed, the intervention period will be prolonged to complete a total of 12 sessions so that patients have an opportunity to receive adequate treatment and derive cumulative benefit. Outcomes will be assessed again at 6 months to evaluate the intermediate effect of intervention, as it is possible that some patients may not receive intensified interventions (including pharmacotherapy) until late in the window for intervention and will likely not have an immediate effect on the outcomes. A number of studies and meta-analyses evaluating long term effect of 8-12 weeks of CBT or collaborative care on depression, fatigue, or pain show sustained benefit at 12 months. [52-56] Whether such sustained effects are seen in this patient population, remains to be evaluated. Thus, we are evaluating the effects of TACcare at 6 months and 12 months to assess the long-term effect of our intervention.

Data Collection and Measures

Patient-Reported Outcomes: Instruments with sound psychometric properties in HD patients have been selected to assess a variety of health domains including the outcomes of interest and potential mediators of outcomes such as sleep quality, anxiety and social support (Table 1). These instruments will be administered centrally by blinded interviewers using computer-assisted telephone interviewing (CATI) which provides high rates of data collection, limits selection bias and minimizes study burden.[57] Self-administered questionnaires may be more difficult to complete for the elderly, minority groups, those with high comorbidity and may exclude patients with low literacy, visual problems and diminished dexterity, creating selection bias.[58] The University

Center for Social & Urban Research (UCSUR) at the University of Pittsburgh will administer CATI through trained interviewers. This approach was used in the Frequent Hemodialysis Network (FHN) Trial and showed high rate of data capture in HD patients.[57] The anticipated time for the assessment is 20-30 minutes. To minimize potential confounding from the effects of the HD procedure, the assessments will be performed on a non-dialysis day at the participant's home. Assessments will be done at baseline, after 12 weeks of intervention (primary outcome) and at 6 and 12-month follow-up.

Measuring Symptom Severity and Variability in Real-time Using Ecological Momentary Assessment (EMA): EMA is a tool to evaluate patient symptoms prospectively and repeatedly in real-time, in the patient's natural environment, and while minimizing respondent burden.[59] Whereas conventional, recall-based questionnaires may fail to capture daily and diurnal variation in symptoms seen in HD patients, resulting in misclassification bias, EMA is well suited to ensure high-fidelity ascertainment of symptoms.[60] Because EMA is well suited to identify those who develop symptoms in a particular context, it could prove useful for recognizing patients who develop post-dialysis fatigue, a debilitating, prevalent, but understudied symptom. [61,62] EMA data collection will be operationalized centrally by UCSUR at the University of Pittsburgh. Subjective fatigue/sleepiness, mood, and cognitive alertness will be assessed using a 19-item questionnaire Daytime Insomnia Symptom Scale (DISS),[63] administered via an interactive voice response system using the patient's cellphone (or a study cellphone if the patient does not have one). Responses on a 7-point Likert scale for each question, with higher scores representing greater endorsement of a particular symptom, will be collected and scored to derive a composite symptom severity score of fatigue, sleepiness and mood. Participants will complete the DISS 4 times daily for 7 consecutive days with each assessment generally requiring 2 to 6 minutes. High patient adherence of over 80% was seen using a similar protocol in our previous study with 55 HD patients completing 1,252 of 1,540 possible assessments, which did not differ by dialysis day.[60] To minimize patient burden, EMA will be conducted only at baseline and at 12 weeks post intervention.

Measuring Treatment Adherence: Treatment adherence will be assessed at baseline, 12-week post intervention and at 6- and 12-month follow-up. *Medication and dietary adherence* will be assessed by using four independent sources: a) self-reported adherence using the 8-item Morisky Medication Adherence Scale (MMAS-8) which also includes items on medication taking behaviors, thus enabling identification of barriers to adherence. This scale is easy to administer, has excellent validity and reliability in patients with chronic conditions, even among those with low literacy.[64] Such self-reported measures have been shown to have moderate to high concordance with objective measures.[65] This scale will be administered centrally by blinded interviewers using computer-assisted telephone interviewing by UCSUR; b) Medication and/or dietary adherence will also be defined by absolute serum phosphorus level <6.0 mg/dL or > 1mg/dL decline in serum phosphorus from preceding month if absolute serum phosphorus level is >6.0 mg/dL, measured as a part of routine clinical care [66,67]; c) Fluid restriction adherence will be determined by inter-dialytic weight gain percentage (of post dialysis weight over preceding one month). Those with inter-dialytic weight gain over3.5% will be classified as non-adherent [68]; d) Medication adherence will also be assessed by verifying pharmacy refill rates for antidepressant, analgesic and antihypertensive medications. *Dialysis adherence* data will be obtained by review of dialysis treatment records. Non-adherence with dialysis will be defined by the percentage of all dialysis sessions skipped and/or requested by the patient to be shortened by ≥ 10 minutes over the 12-week intervention period. Dialysis sessions missed due to hospitalization will not be included as a skipped treatment.

Measuring Selected Pro/anti-inflammatory Cytokines: Selected cytokines *(high sensitivity C - reactive protein; hs-CRP, Interleukin; IL-6, IL-10 and Tumor Necrosis Factor; TNF-α*) were chosen based on their reported associations with the outcomes of interest. [9,16,18,69] All blood samples will be shipped at regular intervals and cytokine measurements will be performed centrally at the University of Pittsburgh. We will collect 10ml of blood drawn pre-dialysis at baseline, post 12-week intervention and at 6-month follow-up. Serum will be aliquoted within 4 hours of the blood draw, shipped on dry ice and stored at -80°C for batch analysis. Cytokine levels will be measuring using Luminex™ bead-set (Millipore Corporation, Billerica MA and Luminex™ 100 IS apparatus, Austin TX) in accordance with the manufacturer's instructions using standardized validated assays and operating procedures.

Descriptors and Potential Covariates: Baseline patient data collection will include socio-demographic data such as age, gender, race, ethnicity, education, marital and employment status, household income, etc. The patient's clinical characteristic such as years on dialysis, comorbid conditions, concomitant medications, etc.

along with data on hospitalizations, mortality and transplantation will be obtained from patient interviews and medical chart abstraction. Routine monthly laboratory assessment done by the dialysis clinics will be extracted.

Adverse Events and Serious Adverse Events
Adverse events (AEs) and serious adverse events (SAEs) will be collected every 2 weeks and whenever the study team learns of an event between the biweekly assessments during the intervention period. These will also be collected at 6- and 12-month follow-up, during which patients will be asked to report all SAE/AEs since the last assessment. The pre-specified serious adverse events include – death, hospitalization/ER visit, bleeding requiring transfusion or hospitalization (due to possible selective serotonin reuptake inhibitor mediated reduction in platelet aggregation), medication overdose requiring ER visit/hospitalization (as determined by review of medical records) and acute suicidal intent. Relatedness of AE/SAEs with study interventions, especially with medications prescribed for symptom management, will be reviewed by external Adverse Events committee and reported.

Side Effects and Tolerability Data
The side effects and tolerability data will be collected every 2 weeks in both study arms. The side effects will be collected using the modified Patient Related Inventory of Side Effects (PRISE) questionnaire.[70] This 20-item questionnaire asks about occurrence and tolerability of 20 common side effects. We will also add an open-ended question to record any other symptoms that patients might have. Although this questionnaire was developed and used to assess side effects of antidepressant medications, we will use the same questionnaire for assessing side effects and tolerability of pain medications because of significant overlap in the commonly reported side effects of these medications.

Outcomes
The primary outcome of the trial is clinically meaningful change (effect size 0.6) in depression, pain or fatigue as ascertained blindly by the *Beck Depression Inventory-II (BDI-II), Functional Assessment of Chronic Illness Therapy Fatigue (FACIT-F)* and *Brief Pain Inventory (BPI) Short form* questionnaires respectively, after a 12-week intervention as compared to control arm. We used effect size (ES) i.e. standardized mean difference instead of raw mean difference as our outcome as this places the emphasis on the most important aspect of an intervention - the size of the effect - rather than its statistical significance. Moreover, in our study the 3 symptoms will be measured using different instruments with varying scales, and ES facilitates standardizing the observed changes in these symptoms. By quantifying the magnitude of the difference between the two study groups, ES gives a more complete and clinically relevant picture of health status change. Cohen's d effect size of 0.6 is considered moderate size and is likely to translate into clinically meaningful improvements in the symptoms.[71] Secondary patient-reported outcomes include change in HRQOL and EMA-measured composite score of fatigue, sleepiness and mood after 12-week intervention. Additional secondary outcomes include adherence to medications, diet and HD treatments; as well as changes in levels of biomarkers of inflammation.

Original Statistical Analysis Plan

Sample Size and Power: We powered our study to detect a clinically meaningful difference in our primary outcomes -- depression, pain, or fatigue -- for Aim 1 at 12 weeks while considering feasibility of subject recruitment. Since there is no validated composite endpoint consisting of the 3 outcomes, we will test the effect of TASCCI on each of the outcomes. We calculated power controlling for False Discovery Rate (FDR) of 5% using the Benjamini-Hochberg correction.[127] To be more conservative, (1) we used a two-sided two-sample t-test in the calculations and (2) assumed that the intervention has a true effect on exactly one endpoint.

Our primary analysis will compare groups at 12 weeks using contrast from a mixed model that includes all time points (baseline, 12 weeks, 6 months, and 12 months). By using all available longitudinal data, our primary analytical approach would be more efficient than t-test and provide greater power to detect differences. We will also have increased power if the intervention has real effect on more than one endpoint.

The projected total sample size is 150, or 75 per group. After accounting for the estimated 10% lost-to-follow-up, the effective total sample size is 136. For this sample size, Table 1 shows the estimated power for a range of effect sizes (standardized mean difference) with and without adjusting for FDR using PASS 13 (NCSS, LLC. Kaysville, UT)

Table 1: Estimated power for total sample size of *N = 136* for a range of effect sizes with and without adjusting for a 5% false discovery rate (FDR).

Effect Size (Standardized Mean Difference)	FDR-unadjusted	FDR-adjusted	
		3 endpoints	4 endpoints
0.4	64%	40%	32%
0.5	82%	70%	63%
**0.6**	**93%**	**88%**	**85%**
0.7	98%	96%	95%
0.8	100%	99%	99%

Thus, our sample size provides 88% power to detect an effect size of 0.6 controlling for 5% FDR for 3 endpoints assuming a true difference exists in 1 out of 3 endpoints. If differences exist on 2 of 3 endpoints, we will have 89% power to detect an effect size of 0.5. The detectable effect size is consistent with what we observed in our study of SCCI in cancer patients (effect size was 0.7 for depression, 0.6 for pain and 0.3 for fatigue, respectively).

In May 2020, the sample size was increased to 160 participants per DSMB recommendations given a higher observed attrition rate (14%).

General Statistical Approach: Distributions of baseline characteristics will be compared between the groups to assess effectiveness of randomization, and statistical differences will be accounted for as covariate in secondary adjusted analyses. All primary analyses will be adjusted for site, age, and comorbidity (design variables) as well as sex and unadjusted analyses will also be reported per CONSORT recommendations. The stratification variables are potentially prognostic of the outcomes, which likely will make the adjusted analyses more efficient than unadjusted. The primary analyses will use the ‘intent-to-treat’ approach in which subjects are analyzed in their assigned group, regardless of treatment adherence, withdrawal or deviation from protocol. General linear mixed models (GLMM) will be used for inferential analyses of primary and secondary outcomes. Unless otherwise specified, all tests will be conducted using two sided 5% level of significance. We will report our findings according to the CONSORT guidelines.

Preliminary Analyses will focus on data checks for completeness and accuracy and address any issues with data quality. The preliminary exploration of the data will be used to: 1) examine univariate and bivariate distributions; 2) identify any imbalances between treatment groups on baseline characteristics; 3) investigate the magnitude of the associations between the dependent variables and the potential covariates; 4) verify the statistical assumptions of the planned primary analyses. If assumptions are violated, then alternative procedures such as data transformations or more robust statistical methods will be considered.

Primary Outcomes Analyses: The three primary patient-reported outcome measures are changes in depression, pain or fatigue as ascertained blindly by the BDI-II, FACIT-F and BPI questionnaires respectively, after a 12-week intervention. Changes in all symptom levels will be assessed and analyzed for each patient. For example, if a patient enters the study because of significant depressive symptoms, we will also assess changes in their levels of fatigue and pain, in addition to changes in depressive symptoms. The rationale for this is that many of these symptoms co-exist, and treatment of one symptom may affect other symptom levels. Also, it may be that patients may not self-perceive some symptoms. For example, if during the course of the intervention, it becomes apparent that patient’s fatigue is more likely a manifestation of underlying depressive symptoms, then they will be offered CBT/medications for depression, in addition to CBT for fatigue. We will use Benjamini-Hochberg correction to maintain a false-discovery-rate of 5% when testing each of the three primary symptom outcomes. [26] for each outcome with fixed treatment group (TĀCcare or health education), time Analyses will use GLMM (baseline, 3, 6, and 12 months), and group by time interaction, adjusting for variables used in the stratified randomization and sex. The inclusion of the random patient intercepts will account for the within-patient correlation due to repeated measures. The group by time interaction captures the treatment effect. This model will be used to test our primary hypothesis of treatment effect after 12 weeks of intervention (immediate effect). As an alternative model that accounts for potential correlation between and allow

simultaneously testing intervention effects across our 3 primary outcomes, we will also fit a joint longitudinal model. This will include the same fixed and random effects terms from the individual models with outcome specific regression coefficients and random effects, unstructured correlation between the random effects, and heterosckedastic residual error terms across the outcomes. In a secondary analysis, we will also test intermediate (6 months) and sustained (12 months) improvements in outcomes. Although we expect patient characteristics to be balanced between groups due to randomization, we will add baseline factors found to be different between groups in secondary adjusted analyses.

Secondary Outcomes Analyses: Our secondary patient-reported outcomes are changes in HRQOL (SF-12 mental and physical component score) in TĀCcare versus control arm after a 12-week intervention. EMA-measured composite symptom severity score will be evaluated at baseline and post-intervention (12 weeks). Adherence outcomes include adherence to medications, diet and dialysis treatments (all continuous variables) measured at 12-weeks, and at 6-and 12-months follow-up. Biomarkers of inflammation include hs-CRP, IL-6, IL-10, and TNF-α measured at baseline, 12-week, and 6-months follow-up. GLMM will also be used to analyze these outcomes. Benjamini-Hochberg correction will be applied to the biomarker analyses. We will also assess the bi-directional association and calculate correlations between the biomarkers and symptoms. Moreover, we will examine how adjusting for the biomarkers influences the TĀCcare effect on the symptoms, and how adjusting for the symptoms influences the effect on the biomarkers.

Subgroup Analyses: The response to treatment interventions may differ among certain patient subgroups. Thus, to explore heterogeneity of intervention effects, we will examine pre-specified subgroups including age – age≤65, age>65; sex – male, female; race – white, black, others; Charlson comorbidity index – less than 4, 4 or more; and dialysis vintage ≤ 1 year, vintage > 1 year. Each variable, its two- and three-way interaction with the treatment group and with time were added to the model, and linear contrasts were used to test for heterogeneity of treatment effects. In addition, we will estimate the treatment effects among those who had elevated symptoms at baseline (FACIT-F≤44, BDI≥16, BPI≥5).

Sensitivity analyses: We will perform additional analyses to assess the robustness and potentially refine our conclusions. This would include assessing intervention as-treated rather than intention-to-treat, nonlinearity of time in the EMA fatigue model, impact of outliers or influential points and impact of missing data.

Intercurrent events (ICE): To maintain a consistent approach, we have selected a hypothetical strategy as our primary approach to handle all ICEs (death, Kidney transplant, switching to home dialysis, patient relocated, withdrawal, treatment dropout/discontinuation, treatment paused due to COVID) to estimate what would have been the outcome at the follow-up time points had the ICE did not occur. Thus, only data that were observed prior to the occurrence of the ICE will be used in fitting mix models for the primary analyses. We opted to keep using mixed models as the primary analytic approach to execute the hypothetical strategy to take advantage of the longitudinal data collection and hence makes more efficient use of available data. This approach assumes post-ICE outcomes can be predicted using observed pre-ICE outcomes and other measured characteristics.

Missing Data: The extent, reasons, randomness of missing data will be examined to identify possible covert missing data mechanisms. Baseline characteristics and intermediate outcomes among those who do or do not complete the study will be compared. The primary analytical approaches (GLMM) can handle data that are ignorably missing (i.e., either missing completely at random or missing at random). However, strategies to handle missing data (single/multiple imputation, selection models, pattern-mixture models, etc.) and sensitivity analyses to encompass different scenarios of assumptions will be considered as appropriate. For measures that were missing due to non-response at the item-level, multiple imputation by chained equations will be used to impute the response at the item level. The median value across 5 imputed datasets will be used in the calculation of overall score.

Summary of Major Revisions to the SAP

1. To gain efficiency, primary analyses adjusted for variables used in stratification as detailed in the design paper (October 2018). Prior to study results unblinding to the study team on August 25, 2022, sex was also added to the model.
2. Subgroup analyses based on charlson comorbidity were made consistent with the cutpoint used in the stratified randomization (CCI<4 and CCI>=4) as detailed in the design paper (October 2018).

Subgroups based on elevated symptom at baseline were also examined to determine if the treatment effects could be different among those who have elevated symptoms at baseline. This was added prior to the study results unblinding to the study team on August 25, 2022.

3. Item-level imputation was performed prior to calculating scores to minimize missing data. This plan was added prior to study results unblinding to the study team on August 25, 2022.
4. To handle intercurrent events (ICE), the estimands approach from the ICH E9 R1 was adopted [Mallinckrodt CH, Bell J, Liu G, Ratitch B, O'Kelly M, Lipkovich I, Singh P, Xu L, Molenberghs G. Aligning Estimators With Estimands in Clinical Trials: Putting the ICH E9(R1) Guidelines Into Practice. Ther Innov Regul Sci. 2020 Mar;54(2):353-364. doi: 10.1007/s43441-019-00063-9. Epub 2020 Jan 6. PMID: 32072593]. This was suggested by an NIDDK biostatistician when the study size was requested to be increased. (September 2020)
5. Due to lack of access to Morisky medication adherence scale which will be used to define the per-protocol set, the per-protocol analyses were not performed.
6. A joint longitudinal model was also used as an alternative model for the three primary outcomes as recommended by the biostatistical reviewer. (March 2023)

Patient-Reported Outcome (PRO) instruments (Appendix)			
**Questionnaire**	**Items**	**Domains**	**Psychometric properties**
Beck Depression Inventory-II (BDI-II)	21	Depression	Excellent internal consistency, test-retest validity and high sensitivity, specificity and responsiveness to treatment in HD population[46,101]
Functional Assessment of Chronic Illness Therapy Fatigue (FACIT-F)	13	Fatigue	Excellent internal consistency, test-retest reliability, responsiveness to interventions and good correlation with SF-36 measured fatigue in HD patients[102,103]
Brief Pain Inventory (BPI) Short form	9	Pain	Extensively used in HD, Excellent construct validity, reliability and responsiveness to treatment[104]
Pittsburgh Sleep Quality Index (PSQI)	19	Sleep quality	Good internal homogeneity, test-retest reliability, discriminant validity, is responsiveness to treatments and has been used in the ESRD population [105,106]
Medical Outcomes Study Short Form-12 (SF-12)	12	Health-related quality of life	The SF-12 scores have excellent intra-class correlation of 0.94 with the SF-36. [107] SF-36 is commonly used in HD patients, and has been extensively tested for reliability and validity in HD patients, well-accepted, responsive to clinical change[57,108]
Multi-dimensional Scale of Perceived Social Support	12	Perceived social support	Has 3 subscales—family, friends, and significant others, has good internal and test-retest reliability and has been used in ESRD patients[109,110]
Generalized Anxiety Disorder-7 (GAD-7) scale	7	Anxiety	Excellent internal consistency, reliability and validity in the general population[111]
NIH Patient Reported Outcomes Measurement Information System (PROMIS) Adult Global Health questionnaire.	29	Physical function, fatigue, pain, emotional distress, sleep	These domains have excellent internal consistency, convergent validity and significant differences between health and disease[113]
Physical Activity Scale for Elderly (PASE)	10	Physical activity	Measures physical activity over last 7 days and has been shown to correlate with accelerometry data in HD patients[114]

References:

1. Abdel-Kader K, Unruh ML, Weisbord SD. Symptom burden, depression, and quality of life in chronic and end-stage kidney disease. *Clinical journal of the American Society of Nephrology : CJASN.* Jun 2009;4(6):1057-1064.
2. Davison SN, Jhangri GS. Impact of pain and symptom burden on the health-related quality of life of hemodialysis patients. *Journal of pain and symptom management.* Mar 2010;39(3):477-485.
3. Jhamb M, Parvez S, Abdel-Kader K, Unruh M, Steel J. Comparison of Prevalence and Predictors of Fatigue in Advanced Chronic Kidney Disease and Cancer patients. *American Society of Nephrology Kidney Week Poster presentation, San Diego, CA.* Nov, 2015.
4. Moens K, Higginson IJ, Harding R, Euro I. Are there differences in the prevalence of palliative care-related problems in people living with advanced cancer and eight non-cancer conditions? A systematic review. *Journal of pain and symptom management.* Oct 2014;48(4):660-677.
5. Cukor D, Peterson RA, Cohen SD, Kimmel PL. Depression in end-stage renal disease hemodialysis patients. *Nature clinical practice. Nephrology.* Dec 2006;2(12):678-687.
6. Davison SN, Koncicki H, Brennan F. Pain in chronic kidney disease: a scoping review. *Seminars in dialysis.* Mar 2014;27(2):188-204.
7. Jhamb M, Weisbord SD, Steel JL, Unruh M. Fatigue in patients receiving maintenance dialysis: a review of definitions, measures, and contributing factors. *American journal of kidney diseases : the official journal of the National Kidney Foundation.* Aug 2008;52(2):353-365.
8. Weisbord SD. Patient-Centered Dialysis Care: Depression, Pain, and Quality of Life. *Seminars in dialysis.* Mar 2016;29(2):158-164.
9. Jhamb M, Argyropoulos C, Steel JL, et al. Correlates and outcomes of fatigue among incident dialysis patients. *Clinical journal of the American Society of Nephrology : CJASN.* Nov 2009;4(11):1779-1786.
10. Jhamb M, Pike F, Ramer S, et al. Impact of fatigue on outcomes in the hemodialysis (HEMO) study. *American journal of nephrology.* 2011;33(6):515-523.
11. Weisbord SD, Fried LF, Arnold RM, et al. Prevalence, severity, and importance of physical and emotional symptoms in chronic hemodialysis patients. *Journal of the American Society of Nephrology : JASN.* Aug 2005;16(8):2487-2494.
12. Belayev LY, Mor MK, Sevick MA, et al. Longitudinal associations of depressive symptoms and pain with quality of life in patients receiving chronic hemodialysis. *Hemodialysis international. International Symposium on Home Hemodialysis.* Apr 2015;19(2):216-224.
13. Cukor D, Rosenthal DS, Jindal RM, Brown CD, Kimmel PL. Depression is an important contributor to low medication adherence in hemodialyzed patients and transplant recipients. *Kidney international.* Jun 2009;75(11):1223-1229.
14. Ghimire S, Castelino RL, Lioufas NM, Peterson GM, Zaidi ST. Nonadherence to Medication Therapy in Haemodialysis Patients: A Systematic Review. *PloS one.* 2015;10(12):e0144119.
15. Weisbord SD, Mor MK, Sevick MA, et al. Associations of depressive symptoms and pain with dialysis adherence, health resource utilization, and mortality in patients receiving chronic hemodialysis. *Clinical journal of the American Society of Nephrology : CJASN.* Sep 5 2014;9(9):1594-1602.
16. Bossola M, Di Stasio E, Giungi S, Rosa F, Tazza L. Fatigue is associated with serum interleukin-6 levels and symptoms of depression in patients on chronic hemodialysis. *Journal of pain and symptom management.* Mar 2015;49(3):578-585.
17. Lopes AA, Bragg J, Young E, et al. Depression as a predictor of mortality and hospitalization among hemodialysis patients in the United States and Europe. *Kidney international.* Jul 2002;62(1):199-207.
18. Taraz M, Taraz S, Dashti-Khavidaki S. Association between depression and inflammatory/anti-inflammatory cytokines in chronic kidney disease and end-stage renal disease patients: a review of literature. *Hemodialysis international. International Symposium on Home Hemodialysis.* Jan 2015;19(1):11-22.
19. Ramkumar N, Beddhu S, Eggers P, Pappas LM, Cheung AK. Patient preferences for in-center intense hemodialysis. *Hemodialysis international. International Symposium on Home Hemodialysis.* Jul 2005;9(3):281-295.
20. Urquhart-Secord R, Craig JC, Hemmelgarn B, et al. Patient and Caregiver Priorities for Outcomes in Hemodialysis: An International Nominal Group Technique Study. *American journal of kidney diseases : the official journal of the National Kidney Foundation.* Mar 8 2016.

21. Davison SN, Levin A, Moss AH, et al. Executive summary of the KDIGO Controversies Conference on Supportive Care in Chronic Kidney Disease: developing a roadmap to improving quality care. *Kidney international.* Sep 2015;88(3):447-459.
22. https://www.cms.gov/Medicare/Quality-Initiatives-Patient-Assessment-Instruments/ESRDQIP/Downloads/PY-2018-Technical-Measure-Specifications.pdf.
23. Koo JR, Yoon JY, Joo MH, et al. Treatment of depression and effect of antidepression treatment on nutritional status in chronic hemodialysis patients. *The American journal of the medical sciences.* Jan 2005;329(1):1-5.
24. Levy NB, Blumenfield M, Beasley CM, Jr., et al. Fluoxetine in depressed patients with renal failure and in depressed patients with normal kidney function. *General hospital psychiatry.* Jan 1996;18(1):8-13.
25. Cukor D, Ver Halen N, Asher DR, et al. Psychosocial intervention improves depression, quality of life, and fluid adherence in hemodialysis. *Journal of the American Society of Nephrology : JASN.* Jan 2014;25(1):196-206.
26. Duarte PS, Miyazaki MC, Blay SL, Sesso R. Cognitive-behavioral group therapy is an effective treatment for major depression in hemodialysis patients. *Kidney international.* Aug 2009;76(4):414-421.
27. Bower JE. Behavioral symptoms in patients with breast cancer and survivors. *Journal of clinical oncology : official journal of the American Society of Clinical Oncology.* Feb 10 2008;26(5):768-777.
28. Xing L, Chen R, Diao Y, Qian J, You C, Jiang X. Do psychological interventions reduce depression in hemodialysis patients?: A meta-analysis of randomized controlled trials following PRISMA. *Medicine.* Aug 2016;95(34):e4675.
29. Barakzoy AS, Moss AH. Efficacy of the world health organization analgesic ladder to treat pain in end-stage renal disease. *Journal of the American Society of Nephrology : JASN.* Nov 2006;17(11):3198-3203.
30. Gielissen MF, Verhagen S, Witjes F, Bleijenberg G. Effects of cognitive behavior therapy in severely fatigued disease-free cancer patients compared with patients waiting for cognitive behavior therapy: a randomized controlled trial. *Journal of clinical oncology : official journal of the American Society of Clinical Oncology.* Oct 20 2006;24(30):4882-4887.
31. Johannsen M, O'Connor M, O'Toole MS, Jensen AB, Hojris I, Zachariae R. Efficacy of Mindfulness-Based Cognitive Therapy on Late Post-Treatment Pain in Women Treated for Primary Breast Cancer: A Randomized Controlled Trial. *Journal of clinical oncology : official journal of the American Society of Clinical Oncology.* Jun 20 2016.
32. Harris TJ, Nazir R, Khetpal P, et al. Pain, sleep disturbance and survival in hemodialysis patients. *Nephrology, dialysis, transplantation : official publication of the European Dialysis and Transplant Association - European Renal Association.* Feb 2012;27(2):758-765.
33. Hedayati SS, Yalamanchili V, Finkelstein FO. A practical approach to the treatment of depression in patients with chronic kidney disease and end-stage renal disease. *Kidney international.* Feb 2012;81(3):247-255.
34. Bower P, Gilbody S, Richards D, Fletcher J, Sutton A. Collaborative care for depression in primary care. Making sense of a complex intervention: systematic review and meta-regression. *The British journal of psychiatry : the journal of mental science.* Dec 2006;189:484-493.
35. Wagner EH, Austin BT, Von Korff M. Organizing care for patients with chronic illness. *The Milbank quarterly.* 1996;74(4):511-544.
36. Davison SN. Pain in hemodialysis patients: prevalence, cause, severity, and management. *American journal of kidney diseases : the official journal of the National Kidney Foundation.* Dec 2003;42(6):1239-1247.
37. Watnick S, Kirwin P, Mahnensmith R, Concato J. The prevalence and treatment of depression among patients starting dialysis. *American journal of kidney diseases : the official journal of the National Kidney Foundation.* Jan 2003;41(1):105-110.
38. Arroll B, Goodyear-Smith F, Crengle S, et al. Validation of PHQ-2 and PHQ-9 to screen for major depression in the primary care population. *Annals of family medicine.* Jul-Aug 2010;8(4):348-353.
39. Norcross JC, Krebs PM, Prochaska JO. Stages of change. *Journal of clinical psychology.* Feb 2011;67(2):143-154.
40. Ju A, Unruh M, Davison S, et al. Establishing a Core Outcome Measure for Fatigue in Patients on Hemodialysis: A Standardized Outcomes in Nephrology-Hemodialysis (SONG-HD) Consensus Workshop Report. *American journal of kidney diseases : the official journal of the National Kidney Foundation.* Jul 2018;72(1):104-112.
41. Trivedi MH. Tools and strategies for ongoing assessment of depression: a measurement-based approach to remission. *The Journal of clinical psychiatry.* 2009;70 Suppl 6:26-31.
42. American Psychiatric Association: Practice Guideline for the Treatment of Patients With Major Depressive Disorder, 3rd Ed., Washington, DC, American Psychiatric Association, 2010.

43. Hofmann SG, Asnaani A, Vonk IJ, Sawyer AT, Fang A. The Efficacy of Cognitive Behavioral Therapy: A Review of Meta-analyses. *Cognitive therapy and research.* Oct 1 2012;36(5):427-440.
44. www.kidneysupportivecare.org/Files/PainBrochure9-09.aspx. Accessed Sept 7th, 2016.
45. Grigoriou SS, Karatzaferi C, Sakkas GK. Pharmacological and Non-pharmacological Treatment Options for Depression and Depressive Symptoms in Hemodialysis Patients. *Health psychology research.* Apr 13 2015;3(1):1811.
46. Cohen SD, Norris L, Acquaviva K, Peterson RA, Kimmel PL. Screening, diagnosis, and treatment of depression in patients with end-stage renal disease. *Clinical journal of the American Society of Nephrology : CJASN.* Nov 2007;2(6):1332-1342.
47. Gale EA. The Hawthorne studies-a fable for our times? *QJM : monthly journal of the Association of Physicians.* Jul 2004;97(7):439-449.
48. Lindquist R, Wyman JF, Talley KM, Findorff MJ, Gross CR. Design of control-group conditions in clinical trials of behavioral interventions. *Journal of nursing scholarship : an official publication of Sigma Theta Tau International Honor Society of Nursing / Sigma Theta Tau.* 2007;39(3):214-221.
49. *Depression: The Treatment and Management of Depression in Adults (Updated Edition). National Collaborating Centre for Mental Health (UK). National Institute for Health and Clinical Excellence: Guidance. Leicester (UK): British Psychological Society; 2010.*
50. Reimherr FW, Amsterdam JD, Quitkin FM, et al. Optimal length of continuation therapy in depression: a prospective assessment during long-term fluoxetine treatment. *The American journal of psychiatry.* Sep 1998;155(9):1247-1253.
51. Steel JL, Geller DA, Kim KH, et al. Web-based collaborative care intervention to manage cancer-related symptoms in the palliative care setting. *Cancer.* Apr 15 2016;122(8):1270-1282.
52. Hensley PL, Nadiga D, Uhlenhuth EH. Long-term effectiveness of cognitive therapy in major depressive disorder. *Depression and anxiety.* 2004;20(1):1-7.
53. Eriksson MCM, Kivi M, Hange D, et al. Long-term effects of Internet-delivered cognitive behavioral therapy for depression in primary care - the PRIM-NET controlled trial. *Scandinavian journal of primary health care.* Jun 2017;35(2):126-136.
54. Li M, Kennedy EB, Byrne N, et al. Systematic review and meta-analysis of collaborative care interventions for depression in patients with cancer. *Psycho-oncology.* May 2017;26(5):573-587.
55. van den Akker LE, Beckerman H, Collette EH, Eijssen IC, Dekker J, de Groot V. Effectiveness of cognitive behavioral therapy for the treatment of fatigue in patients with multiple sclerosis: A systematic review and meta-analysis. *Journal of psychosomatic research.* Nov 2016;90:33-42.
56. Turner JA, Mancl L, Aaron LA. Short- and long-term efficacy of brief cognitive-behavioral therapy for patients with chronic temporomandibular disorder pain: a randomized, controlled trial. *Pain.* Apr 2006;121(3):181-194.
57. Jhamb M, Tamura MK, Gassman J, et al. Design and rationale of health-related quality of life and patient-reported outcomes assessment in the Frequent Hemodialysis Network trials. *Blood purification.* 2011;31(1-3):151-158.
58. Unruh M, Yan G, Radeva M, et al. Bias in assessment of health-related quality of life in a hemodialysis population: a comparison of self-administered and interviewer-administered surveys in the HEMO study. *Journal of the American Society of Nephrology : JASN.* Aug 2003;14(8):2132-2141.
59. Moskowitz DS, Young SN. Ecological momentary assessment: what it is and why it is a method of the future in clinical psychopharmacology. *Journal of psychiatry & neuroscience : JPN.* Jan 2006;31(1):13-20.
60. Abdel-Kader K, Jhamb M, Mandich LA, et al. Ecological momentary assessment of fatigue, sleepiness, and exhaustion in ESKD. *BMC nephrology.* 2014;15:29.
61. Lindsay RM, Heidenheim PA, Nesrallah G, Garg AX, Suri R, Daily Hemodialysis Study Group London Health Sciences C. Minutes to recovery after a hemodialysis session: a simple health-related quality of life question that is reliable, valid, and sensitive to change. *Clinical journal of the American Society of Nephrology : CJASN.* Sep 2006;1(5):952-959.
62. Sklar AH, Riesenberg LA, Silber AK, Ahmed W, Ali A. Postdialysis fatigue. *American journal of kidney diseases : the official journal of the National Kidney Foundation.* Nov 1996;28(5):732-736.
63. Buysse DJ, Thompson W, Scott J, et al. Daytime symptoms in primary insomnia: a prospective analysis using ecological momentary assessment. *Sleep medicine.* Apr 2007;8(3):198-208.

64. Lam WY, Fresco P. Medication Adherence Measures: An Overview. *BioMed research international.* 2015;2015:217047.
65. Garber MC, Nau DP, Erickson SR, Aikens JE, Lawrence JB. The concordance of self-report with other measures of medication adherence: a summary of the literature. *Medical care.* Jul 2004;42(7):649-652.
66. Loghman-Adham M. Medication noncompliance in patients with chronic disease: issues in dialysis and renal transplantation. *The American journal of managed care.* Feb 2003;9(2):155-171.
67. Block GA, Hulbert-Shearon TE, Levin NW, Port FK. Association of serum phosphorus and calcium x phosphate product with mortality risk in chronic hemodialysis patients: a national study. *American journal of kidney diseases : the official journal of the National Kidney Foundation.* Apr 1998;31(4):607-617.
68. Lindberg M, Prutz KG, Lindberg P, Wikstrom B. Interdialytic weight gain and ultrafiltration rate in hemodialysis: lessons about fluid adherence from a national registry of clinical practice. *Hemodialysis international. International Symposium on Home Hemodialysis.* Apr 2009;13(2):181-188.
69. Lasselin J, Kemani MK, Kanstrup M, et al. Low-grade inflammation may moderate the effect of behavioral treatment for chronic pain in adults. *Journal of behavioral medicine.* Oct 2016;39(5):916-924.
70. Bruera E, Yennurajalingam S, Palmer JL, et al. Methylphenidate and/or a nursing telephone intervention for fatigue in patients with advanced cancer: a randomized, placebo-controlled, phase II trial. *Journal of clinical oncology : official journal of the American Society of Clinical Oncology.* Jul 1 2013;31(19):2421-2427.
71. Kazis LE, Anderson JJ, Meenan RF. Effect sizes for interpreting changes in health status. *Medical care.* Mar 1989;27(3 Suppl):S178-189.
72. Kahan BC, Morris TP. Reporting and analysis of trials using stratified randomisation in leading medical journals: review and reanalysis. *BMJ.* Sep 14 2012;345:e5840.
73. Schulz KF, Altman DG, Moher D. CONSORT 2010 statement: updated guidelines for reporting parallel group randomised trials. *BMJ.* Mar 23 2010;340:c332.
74. Lee PH. Covariate adjustments in randomized controlled trials increased study power and reduced biasedness of effect size estimation. *Journal of clinical epidemiology.* Aug 2016;76:137-146.
75. Pocock SJ, Assmann SE, Enos LE, Kasten LE. Subgroup analysis, covariate adjustment and baseline comparisons in clinical trial reporting: current practice and problems. *Statistics in medicine.* Oct 15 2002;21(19):2917-2930.
76. Schulz KF, Altman DG, Moher D. CONSORT 2010 statement: updated guidelines for reporting parallel group randomised trials. *PLoS medicine.* Mar 24 2010;7(3):e1000251.
77. Benjamini Y, Hochberg Y. Controlling the false discovery rate: a practical and powerful approach to multiple testing. *Journal of the Royal Statistical Society, Series B. 57 (1): 289–300.* 1995.
78. Dobscha SK, Corson K, Perrin NA, et al. Collaborative care for chronic pain in primary care: a cluster randomized trial. *Jama.* Mar 25 2009;301(12):1242-1252.
79. Gilbody S, Bower P, Fletcher J, Richards D, Sutton AJ. Collaborative care for depression: a cumulative meta-analysis and review of longer-term outcomes. *Archives of internal medicine.* Nov 27 2006;166(21):2314-2321.
80. Webster LR, Webster RM. Predicting aberrant behaviors in opioid-treated patients: preliminary validation of the Opioid Risk Tool. *Pain Med.* Nov-Dec 2005;6(6):432-442.
81. Farrokhi F, Abedi N, Beyene J, Kurdyak P, Jassal SV. Association between depression and mortality in patients receiving long-term dialysis: a systematic review and meta-analysis. *American journal of kidney diseases : the official journal of the National Kidney Foundation.* Apr 2014;63(4):623-635.
82. Weisbord SD, Fried LF, Mor MK, et al. Renal provider recognition of symptoms in patients on maintenance hemodialysis. *Clinical journal of the American Society of Nephrology : CJASN.* Sep 2007;2(5):960-967.
83. Tonelli M. The roads less traveled? Diverging research and clinical priorities for dialysis patients and those with less severe CKD. *American journal of kidney diseases : the official journal of the National Kidney Foundation.* Jan 2014;63(1):124-132.
84. Donovan DM, Marlatt GA. Recent developments in alcoholism:behavioral treatment. *Recent developments in alcoholism : an official publication of the American Medical Society on Alcoholism, the Research Society on Alcoholism, and the National Council on Alcoholism.* 1993;11:397-411.
85. Von Korff M, Tiemens B. Individualized stepped care of chronic illness. *The Western journal of medicine.* Feb 2000;172(2):133-137.
86. Chiu YW, Teitelbaum I, Misra M, de Leon EM, Adzize T, Mehrotra R. Pill burden, adherence, hyperphosphatemia, and quality of life in maintenance dialysis patients. *Clinical journal of the American Society of Nephrology : CJASN.* Jun 2009;4(6):1089-1096.

87. Weisbord SD, Mor MK, Green JA, et al. Comparison of symptom management strategies for pain, erectile dysfunction, and depression in patients receiving chronic hemodialysis: a cluster randomized effectiveness trial. *Clinical journal of the American Society of Nephrology : CJASN.* Jan 2013;8(1):90-99.
88. Pena-Polanco JE, Mor MK, Tohme FA, Fine MJ, Palevsky PM, Weisbord S. Acceptance of Anti-Depressant Treatment by Patients Receiving Chronic Hemodialysis and Their Renal Providers *(Submitted, under 2nd review).*
89. Jhamb M, Cavanaugh KL, Bian A, et al. Disparities in Electronic Health Record Patient Portal Use in Nephrology Clinics. *Clinical journal of the American Society of Nephrology : CJASN.* Nov 6 2015;10(11):2013-2022.
90. De Las Cuevas C, Arredondo MT, Cabrera MF, Sulzenbacher H, Meise U. Randomized clinical trial of telepsychiatry through videoconference versus face-to-face conventional psychiatric treatment. *Telemedicine journal and e-health : the official journal of the American Telemedicine Association.* Jun 2006;12(3):341-350.
91. O'Reilly R, Bishop J, Maddox K, Hutchinson L, Fisman M, Takhar J. Is telepsychiatry equivalent to face-to-face psychiatry? Results from a randomized controlled equivalence trial. *Psychiatr Serv.* Jun 2007;58(6):836-843.
92. Stenvinkel P, Gillespie IA, Tunks J, et al. Inflammation Modifies the Paradoxical Association between Body Mass Index and Mortality in Hemodialysis Patients. *Journal of the American Society of Nephrology : JASN.* May 2016;27(5):1479-1486.
93. Sun J, Axelsson J, Machowska A, et al. Biomarkers of Cardiovascular Disease and Mortality Risk in Patients with Advanced CKD. *Clinical journal of the American Society of Nephrology : CJASN.* Jul 7 2016;11(7):1163-1172.
94. Cleeland CS, Bennett GJ, Dantzer R, et al. Are the symptoms of cancer and cancer treatment due to a shared biologic mechanism? A cytokine-immunologic model of cancer symptoms. *Cancer.* Jun 1 2003;97(11):2919-2925.
95. Zhang W, He J, Zhang F, et al. Prognostic role of C-reactive protein and interleukin-6 in dialysis patients: a systematic review and meta-analysis. *Journal of nephrology.* Mar-Apr 2013;26(2):243-253.
96. Sin NL, DiMatteo MR. Depression treatment enhances adherence to antiretroviral therapy: a meta-analysis. *Annals of behavioral medicine : a publication of the Society of Behavioral Medicine.* Jun 2014;47(3):259-269.
97. Sutcigil L, Oktenli C, Musabak U, et al. Pro- and anti-inflammatory cytokine balance in major depression: effect of sertraline therapy. *Clinical & developmental immunology.* 2007;2007:76396.
98. Himmerich H, Binder EB, Kunzel HE, et al. Successful antidepressant therapy restores the disturbed interplay between TNF-alpha system and HPA axis. *Biological psychiatry.* Oct 15 2006;60(8):882-888.
99. Bower JE, Lamkin DM. Inflammation and cancer-related fatigue: mechanisms, contributing factors, and treatment implications. *Brain, behavior, and immunity.* Mar 2013;30 Suppl:S48-57.
100. Green JA, Mor MK, Shields AM, et al. Renal provider perceptions and practice patterns regarding the management of pain, sexual dysfunction, and depression in hemodialysis patients. *Journal of palliative medicine.* Feb 2012;15(2):163-167.
101. Hedayati SS, Bosworth HB, Kuchibhatla M, Kimmel PL, Szczech LA. The predictive value of self-report scales compared with physician diagnosis of depression in hemodialysis patients. *Kidney international.* May 2006;69(9):1662-1668.
102. Glaspy JA, Jadeja JS, Justice G, Fleishman A, Rossi G, Colowick AB. A randomized, active-control, pilot trial of front-loaded dosing regimens of darbepoetin-alfa for the treatment of patients with anemia during chemotherapy for malignant disease. *Cancer.* Mar 1 2003;97(5):1312-1320.
103. Jhamb M, Liang K, Yabes J, et al. Prevalence and correlates of fatigue in chronic kidney disease and end-stage renal disease: are sleep disorders a key to understanding fatigue? *American journal of nephrology.* 2013;38(6):489-495.
104. Upadhyay C, Cameron K, Murphy L, Battistella M. Measuring pain in patients undergoing hemodialysis: a review of pain assessment tools. *Clinical kidney journal.* Aug 2014;7(4):367-372.
105. Buysse DJ, Reynolds CF, 3rd, Monk TH, Berman SR, Kupfer DJ. The Pittsburgh Sleep Quality Index: a new instrument for psychiatric practice and research. *Psychiatry Res.* May 1989;28(2):193-213.
106. King AC, Oman RF, Brassington GS, Bliwise DL, Haskell WL. Moderate-intensity exercise and self-rated quality of sleep in older adults. A randomized controlled trial. *Jama.* Jan 1 1997;277(1):32-37.
107. Lacson E, Jr., Xu J, Lin SF, Dean SG, Lazarus JM, Hakim RM. A comparison of SF-36 and SF-12 composite scores and subsequent hospitalization and mortality risks in long-term dialysis patients. *Clinical journal of the American Society of Nephrology : CJASN.* Feb 2010;5(2):252-260.

108. Meyer KB, Espindle DM, DeGiacomo JM, Jenuleson CS, Kurtin PS, Davies AR. Monitoring dialysis patients' health status. *American journal of kidney diseases : the official journal of the National Kidney Foundation.* Aug 1994;24(2):267-279.
109. Kimmel PL, Peterson RA, Weihs KL, et al. Psychosocial factors, behavioral compliance and survival in urban hemodialysis patients. *Kidney international.* Jul 1998;54(1):245-254.
110. Zimet GD, Powell SS, Farley GK, Werkman S, Berkoff KA. Psychometric characteristics of the Multidimensional Scale of Perceived Social Support. *Journal of personality assessment.* Winter 1990;55(3-4):610-617.
111. Lowe B, Decker O, Muller S, et al. Validation and standardization of the Generalized Anxiety Disorder Screener (GAD-7) in the general population. *Medical care.* Mar 2008;46(3):266-274.
112. Edinger JD, Grubber J, Ulmer C, Zervakis J, Olsen M. A Collaborative Paradigm for Improving Management of Sleep Disorders in Primary Care: A Randomized Clinical Trial. *Sleep.* 2016;39(1):237-247.
113. Almutary H, Bonner A, Douglas C. Symptom burden in chronic kidney disease: a review of recent literature. *Journal of renal care.* Sep 2013;39(3):140-150.
114. Johansen KL, Painter P, Kent-Braun JA, et al. Validation of questionnaires to estimate physical activity and functioning in end-stage renal disease. *Kidney international.* Mar 2001;59(3):1121-1127.
