# Supplementary material for: Effects of Technology Assisted Stepped Collaborative Care Intervention to Improve Symptoms in Patients Undergoing Hemodialysis: The TĀCcare Randomized Clinical Trial
Source: JAMA Intern Med. 2023 Jun 20;183(8):795–805. doi: 10.1001/jamainternmed.2023.2215 (PMC10282960; doi:10.1001/jamainternmed.2023.2215)
Supplement: Supplement 2. — eMethods. Statistical Analysis Plan eFigure. Distribution of patient symptoms at baseline eTable 1. Symptom scores using validated questionnaires at baseline among those with fatigue, pain or depression at screening eTable 2. Baseline levels of other patient-reported outcomes and adherence to medications, diet, fluid restriction and hemodialysis treatments eTable 3. Summary of telemedicine sessions for TĀCcare study participants eTable 4a. Change in proportion of patients reporting using antidepressant or analgesic medications from baseline to 3-months eTable 4b. Change in average medication dose from baseline to 3-months among participants reporting use at baseline eTable 5. Adverse events by TĀCcare study group [file jamainternmed-e232215-s002.pdf]

Supplemental Online Content

Jhamb M, Steel JL, Yabes JG, et al. Effects of a technology assisted stepped collaborative care intervention to improve symptoms in patients undergoing hemodialysis: the TĀCcare randomized clinical trial. *JAMA Intern Med*. Published June 20, 2023. doi:10.1001/jamainternmed.2023.2215

eFigure 1. Distribution of patient symptoms at baseline

eTable 1. Symptom scores using validated questionnaires at baseline among those with fatigue, pain or depression at screening

eTable 2. Baseline levels of other patient-reported outcomes and adherence to medications, diet, fluid restriction and hemodialysis treatments

eTable 3. Summary of telemedicine sessions for TĀCcare study participants

eTable 4a. Change in proportion of patients reporting using antidepressant or analgesic medications from baseline to 3-months

eTable 4b. Change in average medication dose from baseline to 3-months among participants reporting use at baseline

eTable 5. Adverse events by TĀCcare study arm

This supplemental material has been provided by the authors to give readers additional information about their work.

© 2023 Jhamb M et al. *JAMA Intern Med*

eFigure 1. Distribution of patient symptoms at baseline

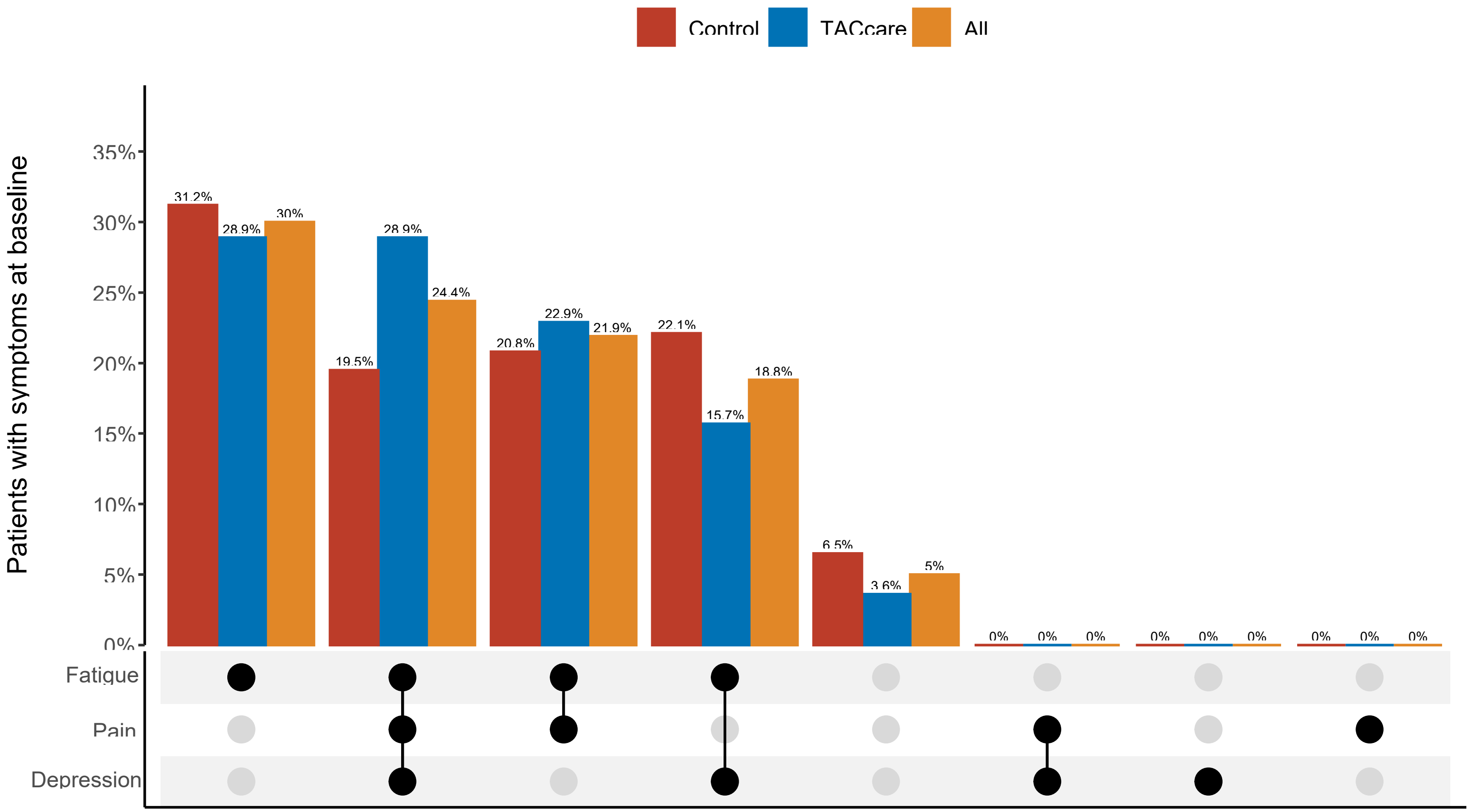

© 2023 Jhamb M et al. *JAMA Intern Med*

eTable 1: Symptom scores using validated questionnaires at baseline among those with fatigue, pain or depression at screening

Screening	Baseline		All (N=160)
Fatigue			136 (85.0%)
	FACIT-F fatigue score	Mean (SD)	27.0 (10.9)
		Median [Min-Max]	28.0 [3.0-48.0]
	Fatigue (FACIT<=44)		131 (96.3%)
Pain			117 (73.1%)
	BPI average pain score	Mean (SD)	4.6 (3.1)
		Median [Min-Max]	5.0 [0.0-10.0]
	Moderate-severe pain (BPI>=5)		69 (59.0%)
Depression			67 (41.9%)
	BDI depression score	Mean (SD)	21.0 (8.7)
		Median [Min-Max]	20.0 [4.0-47.0]
	BDI depression score (BDI>=16)		49 (73.1%)

© 2023 Jhamb M et al. *JAMA Intern Med*

eTable 2: Baseline levels of other patient-reported outcomes and adherence to medications, diet, fluid restriction and hemodialysis treatments

Characteristic	TACcare (n=83)	Control (n=77)
**BPI Pain Interference Score**	3.7 (3.2)	3.4 (3.3)
**BPI Pain severity score**	3.7 (3.1)	3.2 (2.9)
**SF-12 PCS**	34.9 (7.4)	34.7 (9.2)
**SF-12 MCS**	38.6 (9.1)	40.8 (8.2)
**GAD Anxiety Score**	6.5 (5.1)	5.5 (4.4)
**Multidimensional Scale of Perceived Social support**	63.6 (14.5)	62.6 (13.7)
**PROMIS fatigue**	58.2 (8.1)	57.7 (9.5)
**PROMIS depression**	54.2 (9.1)	52.4 (8.8)
**PROMIS pain interference**	59.4 (8.5)	60.0 (10.5)
**PSQI Sleep Score**	9.2 (3.6)	8.7 (3.2)
**PASE Physical activity**	225.9 (52.7)	234.2 (55.6)
**Medication/dietary adherent***	58 (69.9%)	51 (66.2%)
**Fluid restriction adherent$ (IDWG% ≤3.5%)**	65 (78.3%)	63 (81.8%)
**Number of HD treatments missed in last 30 days**	1.0 (1.7)	0.8 (1.3)

BPI Pain interference score -average of 7 items on pain interference in BPI; BPI Pain severity score: average of 4 pain severity items in BPI; SF-12: Medical Outcomes Study Short Form-12 (SF-12), PCS: physical component score, MCS: mental component score; PROMIS: NIH Patient Reported Outcomes Measurement Information System Adult Global Health; PSQI: Pittsburgh Sleep Quality Index; GAD: Generalized Anxiety Disorder-7); PASE: Physical Activity Scale for Elderly

*Adherence defined as serum phosphorus level <6.0 mg/dL or > 1mg/dL decline in serum phosphorus from preceding month if absolute serum phosphorus level is >6.0 mg/dL

$IDWG% - inter-dialytic weight gain percentage (of post dialysis weight over preceding 1 month).

© 2023 Jhamb M et al. *JAMA Intern Med*

eTable 3: Summary of telemedicine sessions for TĀCcare study participants

	**TACCare (n=83)**	**Attention-Control (n=77)**
Number of sessions completed	667	435
Avg. length of sessions	46.3 min	27.0 min
% of patients with >=80% session completed [10 TACCare sessions or 5 attention control sessions]	48 (80.0%)	71 (94.7%)
% of patients with 50-80% session completed [6-9 TACCare sessions or 3-4 attention control sessions]	8 (13.3%)	2 (2.7%)
% of patients with<50% session completed [<6 TACCare sessions or <3 attention control sessions]	4 (6.7%)	2 (2.7%)

© 2023 Jhamb M et al. *JAMA Intern Med*

eTable 4a. Change in proportion of patients reporting using antidepressant or analgesic medications from baseline to 3-months

Variable		All	TACcare	Control	P
	N	134	60	74	
Antidepressant use	Never users	81 (60.4%)	32 (53.3%)	49 (66.2%)	0.063
	Incident users	7 (5.2%)	2 (3.3%)	5 (6.8%)	
	Discontinued use	5 (3.7%)	1 (1.7%)	4 (5.4%)	
	Prevalent users	41 (30.6%)	25 (41.7%)	16 (21.6%)	
Opioid use	Never users	88 (65.7%)	40 (66.7%)	48 (64.9%)	0.867
	Incident users	4 (3.0%)	2 (3.3%)	2 (2.7%)	
	Discontinued use	7 (5.2%)	2 (3.3%)	5 (6.8%)	
	Prevalent users	35 (26.1%)	16 (26.7%)	19 (25.7%)	
Opioid or Analgesic use	Never users	33 (24.6%)	15 (25.0%)	18 (24.3%)	0.336
	Incident users	9 (6.7%)	3 (5.0%)	6 (8.1%)	
	Discontinued use	7 (5.2%)	1 (1.7%)	6 (8.1%)	
	Prevalent users	85 (63.4%)	41 (68.3%)	44 (59.5%)	

*restricted to participants who were not loss to follow-up at 3-months
Never users: Participants who were not on medications from baseline to 3-months follow-up
Incident users: Participants who started a new medication during 3-months follow-up
Discontinued use: Participants who discontinued a medication during 3-months follow-up
Prevalent users: Participants who continued the medication during 3-months follow-up

eTable 4b. Change in average medication dose from baseline to 3-months among participants reporting use at baseline

Medication*			TACcare	Control	
		N(TACcare)	mean(sd)	mean(sd)	N(Control)
Citalopram (mg/d)	Baseline	3	20(0)	22.5(12.6)	4
	3 months	3	20(0)	25(10)	4
	Change	3	0(0)	2.5(5)	4
Paroxetine (mg/d)	Baseline	2	25(21.2)	20(NA)	1
	3 months	2	25(21.2)	20(NA)	1
	Change	2	0(0)	0(NA)	1

© 2023 Jhamb M et al. *JAMA Intern Med*

Sertraline (mg/d)	Baseline	5	80(44.7)	79.1(33.2)	6
	3 months	5	80(44.7)	87.5(44)	6
	Change	5	0(0)	8.3(20.4)	6
Wellbutrin (mg/d)	Baseline	4	225(95.7)	250(70.7)	2
	3 months	4	225(59.7)	300(0)	2
	Change	4	0(0)	50(70.7)	2
Opioid dose (MME/d)	Baseline	12	24.5(22.9)	24(21.5)	15
	3 months	12	23.2(15.5)	36.4(25.6)	15
	Change	12	2.72(8.6)	12.3(26.6)	15

* Only 4 most frequently reported antidepressants shown

© 2023 Jhamb M et al. *JAMA Intern Med*

eTable 5: Adverse events by TĀCcare study arm

Body System and Preferred Term*	Total consented patients (n=215)	Control n=57	Intervention n=55
	Number of events		
**Cardiovascular Disease**	**83**	**39**	**40**
Hypotension/Hypertension	11	2	8
Chest pain/CAD	23	10	11
Volume overload/dyspnea/CHF decompensation	29	15	13
Other	20	12	8
**Infectious**	**74**	**36**	**35**
**Neurological**	**12**	**2**	**9**
**Pulmonary**	**4**	**2**	**2**
**Peripheral Vascular Disease**	**7**	**3**	**3**
Amputation	3	3	0
Other	4	0	3
**Dialysis Access Related**	**43**	**19**	**22**
Fistula creation/complication	20	9	11
Infiltration	6	0	6
Malfunction/complication	17	10	5
**Gastrointestinal**	**30**	**19**	**11**
Abdominal pain/nausea/vomiting/diarrhea	14	8	6
Bowel obstruction	4	3	1
Other	12	8	4
**Others**	**104**	**53**	**50**
Bleeding	5	5	0
Fall			
Primary	16	10	5
Secondary	3	2	1
Fracture			
Primary	9	6	3
Secondary	5	3	2
Hyperkalemia	9	2	7
Kidney transplant surgery	5	1	3
Missed Dialysis	2	0	2
Psychological	3	2	1
Pain	16	5	11
Sub-therapeutic INR	1	1	0
Miscellaneous	31	17	13
**Total**	**357**	**173**	**172**

© 2023 Jhamb M et al. *JAMA Intern Med*
