# Supplementary material for: Effects of Technology Assisted Stepped Collaborative Care Intervention to Improve Symptoms in Patients Undergoing Hemodialysis: The TĀCcare Randomized Clinical Trial
Source: JAMA Intern Med. 2023 Jun 20;183(8):795–805. doi: 10.1001/jamainternmed.2023.2215 (PMC10282960; doi:10.1001/jamainternmed.2023.2215)
Supplement: Supplement 3. — Data Sharing Statement [file jamainternmed-e232215-s003.pdf]

Data Sharing Statement

Jhamb. Effects of Technology-Assisted Stepped Collaborative Care Intervention to Improve Symptoms in Patients Undergoing Hemodialysis. *JAMA Intern Med*. Published June 20, 2023. doi:10.1001/jamainternmed.2023.2215

Data
Data available: Yes
Data types: Deidentified participant data, Data dictionary
How to access data: Will be provided once requested
When available: beginning date: 01-01-2024, end date: 12-31-2025

Supporting Documents
Document types: None

Additional Information
Who can access the data: The following data will be made available beginning Jan 1st 2024 and ending Dec 31st, 2025: deidentified participant data and data dictionary (contact Manisha Jhamb; e-mail, jhambm@upmc.edu). These data will be made available to investigators with a methodologically sound proposal, with institutional review board approval and demonstration of resources to be able to undertake the proposed analyses, for a wide range of purposes subject to review and approval by the study's executive committee, and after approval of the proposal, consistent with guidelines of the University of Pittsburgh.
Types of analyses: as above
Mechanisms of data availability: as above
